# BaeR and H-NS control CRISPR-Cas-mediated immunity and virulence in *Acinetobacter baumannii*

**DOI:** 10.1128/msystems.01067-25

**Published:** 2025-10-31

**Authors:** Ting Yu, Jun Xie, Xinyue Huang, Jiayuan Huang, Guangyu Bao, Wenjie Yuan, Chengfeng Gao, Cuicui Liu, Jian Hu, Weixuan Yang, Guocai Li

**Affiliations:** 1 The First School of Clinical Medicine, Faculty of Medicine, Yangzhou University 38043, Yangzhou, China; 2 Department of Laboratory Medicine, Xuyi People's Hospital/Clinical College, Yangzhou University 38043, Huai’an, China; 3 School of Basic Medical Sciences & School of Public Health, Faculty of Medicine, Yangzhou University 38043, Yangzhou, China; 4 Department of Laboratory Medicine, Affiliated Hospital, Yangzhou University 38043, Yangzhou, China; 5 Department of Laboratory Medicine, Yixing Hospital of Traditional Chinese Medicine, Guangling College, Yangzhou University 38043, Yangzhou, China; 6 The Fifth People’s Hospital of Huai’an/Huai’an Hospital Affiliated to Yangzhou University 38043, Huai’an, China; 7 The Key Laboratory of the Jiangsu Higher Education Institutions for Nucleic Acid & Cell Fate Regulation (Yangzhou University) 38043, Yangzhou, China; 8 Jiangsu Key Laboratory of Zoonosis/Jiangsu Co-Innovation Center for Prevention and Control of Important Animal Infectious Diseases and Zoonoses, Yangzhou University 38043, Yangzhou, China; Pacific Northwest National Laboratory, Richland, Washington, USA

**Keywords:** *Acinetobacter baumannii*, CRISPR-Cas, BaeR, H-NS, immunity, biofilm, virulence

## Abstract

*Acinetobacter baumannii* balances its remarkable ability to acquire antibiotic resistance genes via horizontal gene transfer (HGT) with the immune defense functions of its CRISPR-Cas system, forming a dynamic equilibrium governed by intricate transcriptional regulation. However, the regulatory mechanisms underlying the I-Fb CRISPR-Cas system in *A. baumannii* remain poorly understood. This study elucidated a multitiered regulatory axis mediated by BaeR and H-NS that coordinates immune defense and virulence expression in the I-Fb CRISPR-Cas system. Using DNA pull-down and electrophoretic mobility shift assay (EMSA), we demonstrated that H-NS directly binds AT-rich regions within the *cas3* promoter, suppressing both interference activity and adaptive immunity of the I-Fb CRISPR-Cas system. Intriguingly, the two-component regulator BaeR controlled this suppression by positively regulating H-NS expression. The results revealed that Δ*cas3* mutants exhibited increased biofilm thickness, elevated the extracellular matrix component poly N-acetyl glucosamine (PNAG) production, upregulated pilus expression, and significantly enhanced epithelial cell adhesion. Strikingly, Δ*h-ns-cas3* and Δ*baeR-cas3* double-knockout strains showed no statistically significant differences in virulence phenotypes compared to the Δ*cas3* single mutants. These findings indicate CRISPR-Cas-mediated inhibition of biofilm formation is abolished upon *cas3* deletion, thereby releasing the regulatory constraints imposed by BaeR and H-NS. This dysregulation leads to excessive biofilm and extracellular matrix component accumulation, ultimately amplifying bacterial colonization capacity and pathogenicity in host environments. This discovery reveals the dual regulatory roles of BaeR and H-NS in the *A. baumannii* I-Fb CRISPR-Cas system, mediating both immune defense and virulence modulation. These insights establish a theoretical foundation for novel antimicrobial strategies targeting CRISPR-Cas regulatory networks.

**IMPORTANCE:**

*A. baumannii*, a leading cause of drug-resistant nosocomial infections, evolves antibiotic resistance through horizontal gene transfer (HGT) while employing CRISPR-Cas systems to limit foreign DNA invasion. This study reveals that the I-Fb CRISPR-Cas system, typically a defense mechanism, functions as a repressor of virulence traits in *A. baumannii*. We demonstrate that the transcriptional regulators H-NS and BaeR form a hierarchical axis suppressing Cas3 expression, thereby constraining biofilm formation and host adhesion. Strikingly, CRISPR-Cas deficiency enhances virulence, thickens biofilms, elevates PNAG production, and enhances epithelial colonization through escape from BaeR-/H-NS-mediated control. This work redefines CRISPR-Cas as a dual-function module balancing immune defense and pathogenicity, exposing the BaeR-H-NS-Cas3 axis as a druggable target for novel anti-infectives aimed at disrupting bacterial adaptive evolution.

## INTRODUCTION

Antimicrobial resistance (AMR) is currently one of the most pressing crises in global health, emerging as a leading cause of death worldwide (1), with projections suggesting 10,000,000 annual AMR-related deaths by 2050 (2). Notably, *A. baumannii* stands out as a significant cause of hospital infections, responsible for approximately 250,000 AMR-related deaths annually due to its rapid evolution of multidrug resistance (MDR) and even pan-resistance (3). This has become a major threat to global public health.

*A. baumannii* predominantly acquires drug resistance genes through horizontal gene transfer (HGT) (4). Mobile genetic elements, such as plasmids, transposons, and integrons, significantly facilitate the dissemination of these drug resistance genes (5). These elements enable the transfer of resistance traits among strains via mechanisms like conjugation and transformation, exacerbating AMR’s emergence and complicating infection prevention and control in healthcare settings (4). In contrast, the innate adaptive immune system in prokaryotes, the CRISPR-Cas system, plays a crucial role in inhibiting HGT and maintaining genomic stability by recognizing and cleaving exogenous nucleic acids, including bacteriophages and plasmids (6–8).

The CRISPR-Cas system consists of a clustered regularly interspaced short palindromic repeats (CRISPR) array and CRISPR-associated (Cas) proteins (9). The CRISPR array consists of repeat sequences and spacer regions that provide a genetic memory bank of past infections (10, 11). Cas proteins are usually encoded near the CRISPR array and participate in different stages of the CRISPR-Cas immunity (12). The CRISPR-Cas immunity includes adaptation (the acquisition of foreign sequences into a CRISPR array), expression (facilitating the maturation of crRNAs), and interference stages (crRNA-guided Cas proteins to target cleavage of invading nucleic acids) (13). In *A. baumannii*, the CRISPR-Cas system protects against phage infections and limits the acquisition of AMR genes (14, 15). However, its functionality is constrained by several factors. First, the expression of CRISPR-Cas demands substantial energy, and it imparts non-negligible fitness costs on the host (16, 17), such as the risk of autoimmunity (18) and repulsion to exogenous beneficial genes (19, 20). These costs can lead to selective loss of the CRISPR-Cas system (21). Second, phages can further undermine CRISPR-Cas defenses by encoding anti-CRISPR proteins (Acr) or evading immune detection through synergistic infection (22, 23).

Recent research has revealed that the stability of CRISPR-Cas systems is controlled by regulatory factors (24–28). The histone-like (H-NS) plays a crucial role in maintaining system quiescence by silencing the transcription of *cas* genes (25). Meanwhile, transcription factors like LeuO and StpA counteract H-NS’s inhibition, activating CRISPR-Cas in response to phage infections or DNA invasions (26). Additionally, global regulators such as cAMP-CRP and the BaeSR two-component system dynamically balance the needs for horizontal gene transfer ( (29, 30). These findings highlight a potential strategy: targeting the regulatory network of CRISPR-Cas could enhance phage therapy effectiveness and help curb the spread of resistance genes. We can play a role in activating the endogenous CRISPR-Cas system, which could work in tandem with phage lysis to specifically eliminate MDR strains, while inhibiting HGT may reduce the dissemination of resistance genes within bacterial populations.

However, several key bottlenecks remain in the research on CRISPR-Cas systems in *A. baumannii*: (1) the regulators of the I-Fb CRISPR-Cas system and their interaction networks have not been fully characterized (ii), how these regulators respond to environment stress and impact system stability requires further investigation; (iii) the feasibility and safety of engineered regulatory strategies, such as small-molecule activators or synthetic biology circuits, need urgent validation.

To address these challenges, this study aims to first identify the regulatory factors involved in the *A. baumannii* I-Fb CRISPR-Cas system and to elucidate their molecular mechanisms.

In this study, we used a clinically isolated strain of *A. baumannii* carrying a complete I-Fb CRISPR-Cas system. The DNA pull-down and electrophoretic mobility shift assay (EMSA) confirmed H-NS direct binding to the *cas3* promoter. Subsequently, we demonstrated that H-NS represses I-Fb CRISPR-Cas-mediated immunity function. Intriguingly, while EMSA revealed partial binding of BaeR to the *cas3* promoter, BaeR suppresses I-Fb CRISPR-Cas activity through H-NS. In addition to its immunity function, the CRISPR-Cas system can also regulate virulence. Cas3 could inhibit the biofilm and extracellular matrix component, poly N-acetyl glucosamine (PNAG). Concurrently, qRT-PCR identified Cas3-dependent downregulation of pilus-associated genes, which correlated with impaired cellular adhesion and attenuated virulence in epithelial colonization assays. To dissect the interplay between BaeR, H-NS, and Cas3 in virulence modulation, we constructed Δ*h-ns-cas3* and Δ*baeR-cas3* double-knockout strains. Strikingly, both double mutants exhibited virulence phenotypes indistinguishable from the Δ*cas3* single mutant, indicating that BaeR and H-NS modulate virulence exclusively through Cas3-dependent mechanisms in I-Fb CRISPR-Cas *A. baumannii*. Collectively, these findings establish a dual regulatory paradigm: not only control CRISPR-Cas immunity but also finetune bacterial virulence via Cas3-mediated suppression of biofilm/EPS production and pilus expression.

## MATERIALS AND METHODS

### Bacterial strains, growth conditions, and antibiotics

The bacterial strains used in the study are listed in Table 1, and all primers and restriction enzymes used in the study are listed in Table S1. The strains were grown in Luria-Bertani (LB) broth/agar at 37°C with shaking at 200 rpm unless otherwise noted. The media were supplemented with antibiotics at the following concentrations: carbenicillin 100 mg/mL (C8251, Solarbio, Beijing, China), kanamycin 50 µg/mL (K8020, Solarbio, Beijing, China), and tetracycline 10 µg/mL (T8180, Solarbio, Beijing, China).

**TABLE 1:** Strains and plasmids^*a*^

Strain or plasmid	Relevant genotype and property	Source and/or reference
Strains		
*A. baumannii* strain AB43	Wild-type	(31)
*ΔCas3*	AB43 deleting *cas3*	(31)
*E.coil* DH5α	Clone host strain	Laboratory stock
BL21 (DE3)	Expression strain	Laboratory stock
BL21-groEL	Bl21 (DE3) carrying pET30a-groEL	This study
BL21-H-NS	Bl21 (DE3) carrying pET30a-H-NS	This study
BL21-BaeR	Bl21 (DE3) carrying pET30a-BaeR	This study
*ΔBaeR*	AB43 deleting *BaeR*	This study
*ΔBaeR*/*pBaeR*	AB43D*BaeR* carrying pBaeR	This study
*ΔH-NS*	AB43 deleting *H-NS*	This study
*ΔH-NS*/*pH-NS*	AB43*ΔH-NS* carrying pH-NS	This study
*ΔH-NS-BaeR*	AB43 deleting *H-NS* and *BaeR*	This study
*ΔH-NS-BaeR*/*pH-NS-BaeR*	AB43*ΔH-NS-BaeR* carrying pH-NS-BaeR	This study
*ΔH-NS-Cas3*	AB43 deleting *H-NS* and *Cas3*	This study
*ΔBaeR-Cas3*	AB43 deleting *BaeR* and *Cas3*	This study
*ΔCsuAB*	AB43 deleting *CsuAB*	This study
ΔC*suAB/pCsuAB*	AB43*ΔCsuAB* carrying pCsuAB	This study
*ΔPilA*	AB43 deleting *PilA*	This study
*ΔPilA/pPilA*	AB43*ΔPilA* carrying pPilA	This study
PCas3-1	AB43 carrying Cas3-1	This study
PCas3-2	AB43 carrying Cas3-2	This study
PCas3-3	AB43 carrying Cas3-3	This study
Plasmids		
pKD4	Kan^r^	(32)
pAT03	pMMB67EH with FLP recombinase	(32)
pAT04	pMMB67EH with Rec_Ab_ system, Tet^r^	(32)
pET30a	Expression vector carrying His tag; Kan^r^	Laboratory stock
pET30a-groEL	pET30a carrying the AB43 *groEL* gene	This study
pET30a-H-NS	pET30a carrying the AB43 *H-NS* gene	This study
pET30a-BaeR	pET30a carrying the AB43 *BaeR* gene	This study
pWH1266	*Acinetobacter* plasmid	Laboratory stock
pBR322-Tac	Amp^r^ and Tet^r^	Laboratory stock
pBRAB	pBR322-Tac carrying origin of replication from plasmid pWH1266	This study
pBBR1MCS-Tac-EGFP	Kan^r^	Laboratory stock
pBEAE	pBBR1MCS-Tac-EGFP carrying origin of replication from plasmid pWH1266	This study
pH-NS	pBRAB carrying *H-NS* with the native promoter	This study
pBaeR	pBRAB carrying *BaeR* with the native promoter	This study
pH-NS-BaeR	pBRAB carrying *H-NS* and *BaeR* with the native promoter	This study
pCsuAB	pBRAB carrying *CsuAB* with the native promoter	This study
pPilA	pBRAB carrying *PilA* with the native promoter	This study
Cas3-1	pBEAB carrying *Cas3* promoter (-163 to 0)	This study
Cas3-2	pBEAB carrying *Cas3* promoter (-297 to 0)	This study
Cas3-3	pBEAB carrying *Cas3* promoter (-425 to 0)	This study
CR-sp20	pBRAB containing the protospacer to CR-sp20	This study
CR-sp50	pBRAB containing the protospacer to CR-sp50	This study
CR-sp75	pBRAB containing the protospacer to CR-sp75	This study
CR-sp20 M	pBRAB containing the protospacer to CR-sp20 with a one-base seed mutation	This study
CR-sp50 M	pBRAB containing the protospacer to CR-sp50 with a one-base seed mutation	This study
CR-sp75 M	pBRAB containing the protospacer to CR-sp75 with a one-base seed mutation	This study

^*a*^ Ampr, ampicillin-resistant; Kanr, kanamycin-resistant, Tetr, tetracycline-resistant.

### Plasmid constructions

The plasmids used in the study are listed in Table 1, and primers are listed in Table S1. The pBRAB plasmid was generated as follows (32). The origin of replication from plasmid pWH1266 was PCR-amplified by KOD DNA Polymerase (KFX-101, Toyobo, Japan) and cloned into the SpeI site of pBR322-Tac by T4 DNA Ligase (2011A, Takara, Japan). Similarly, the pBEAB plasmid was constructed. The origin of replication was amplified and ligated to pBBR1MCS-Tac-EGFP (PvuI). The pH-NS, pBaeR, pH-NS-BaeR, CR-sp20, CR-sp20 M, CR-sp50, CR-sp50 M, CR-sp75, and CR-sp75 M plasmids were constructed as follows. The *h-ns*, *baeR, h-ns-baeR,* and the different target spacer sequences genes were amplified and ligated to plasmid pBRAB. The PCas3-1, PCas3-2, and PCas3-3 plasmids were constructed as follows. The different *cas3* promoters were amplified and ligated to plasmid pBEAB. The pET30a-groEL, pET30a-H-NS, and pET30a-BaeR plasmids were constructed as follows. The fragments of *groEL*, *h-ns*, and *baeR* genes were amplified and ligated to pET30a.

### Cell lines and growth conditions

The A549 cell line was obtained from the Department of Microbiology, Institute of Translational Medicine, Medical College, Yangzhou University. The cells were cultured in RPMI 1640 medium (G4531, Servicebio, Wuhan, China) containing 10% heat-inactivated fetal bovine serum (FBS) (C8500, NCM Biotech, Suzhou, China) and 1% penicillin-streptomycin (C0222, Beyotime, Shanghai, China), at 37°C in a 5% CO_2_ incubator.

### DNA pull-down assays

DNA pull-down assays were performed according to the instructions (Bes5004, BersinBio, Guangzhou, China). The biotin-labeled and unlabeled PCR primers were synthesized (Takara, Beijing, China) (Table S1). Briefly, the positive biotin-labeled sequence (−297 to 0 of *cas3*) was amplified from AB43 genomic DNA and purified, respectively. The DNA was incubated with washed magnetic beads. Next, the proteins were incubated with magnetic bead probes, and the beads were subsequently washed three times to remove non-adhering and low-specificity DNA-binding proteins. The samples were eluted with buffer and collected for SDS-PAGE and silver staining (P0017S, Beyotime, Shanghai, China) and then analyzed by mass spectrometry (MS).

### Analysis of the *cas3* promoter activity

The *cas3* promoter activity was carried out as a previous study (33). Briefly, the bacterial strains were diluted to 5 × 10^8^ CFU/mL and 1:100 into 2 mL LB broth. After incubation in a stationary phase at 37°C, the cells were normalized by the OD600, washed three times with PBS, and resuspended in an equal volume of PBS. The fluorescence was recorded using a Tecan Spark 10M microtiter reader (excitation λ of 395 nm; emission λ of 507 nm).

### Protein expression and purification

All recombinant proteins have an N-terminal 6 × His tag, and all proteins were overproduced in *E. coli* BL21 (DE3) (Weidibio, Shanghai, China) grown in LB broth in the presence of 50 µg/mL kanamycin. After the optical density reached 0.6 at 600 nm (OD600), cells were induced with 1 mM β-D-1-thiogalactopyranoside (IPTG) (I8070, Solarbio, Beijing, China) for 6 h, and cells were harvested and frozen at –80°C. Cell pellets were resuspended in lysis buffer [50 mM NaH_2_PO_4_, 300 mM NaCl, pH 8.0] and broken by ultrasound and centrifugation at 4℃. His_6_-tagged proteins were purified by a high-affinity nickel-charged nitrilotriacetic acid (Ni–NTA) resin (P2233, Beyotime, Shanghai, China). For protein purification, affinity resins were washed with lysis buffer before elution. His_6_-tagged proteins were eluted with elution buffer [50 mM NaH_2_PO_4_, 300 mM NaCl, 250 mM imidazole, pH 8.0]. After dialysis by PBS, purified proteins were stored at –80°C. SDS-PAGE and Coomassie Brilliant Blue PAGE staining (abs9750, absin, Shanghai, China) analyzed the purified protein.

### Electrophoretic mobility shift assays

The EMSAs were carried out as in a previous study (34). DNA probes were amplified and purified for EMSAs. In a 10 µL reaction system, 0.1 pmol of DNA probes was mixed with proteins in binding buffer [20 mM HEPES, 10 mM (NH_4_)_2_SO_4_, 1 mM DTT, 30 mM KCl, 1% Tween 20, 1 mM EDTA, pH 7.6] for 25°C at 30 min. The reaction mixtures were then subjected to a 6% native polyacrylamide gel and run in the 0.5 × TBE buffer at 100 V for 50 min. The gel was stained in 0.5 × TBE buffer containing 1 × SYBR Safe DNA gel stain (GR501, Vazyme, Nanjing, China) and imaged by the Tanon gel analysis software version 2.30 (Tanon 5200Multi, China). The nonspecific competitor probe was the same as for the EMSA described above. Competitive EMSAs were performed by incubating first recombinant H-NS (rH-NS) with DNA probes for 10 min, followed by the subsequent addition of recombinant BaeR (rBaeR) and incubation for another 20 min. In addition, for competitive binding experiments, the reactions were also performed by incubating first rBaeR with DNA probes for 10 min, followed by the subsequent addition of rH-NS and incubation for another 20 min.

### Western blot analysis

The mid-log phase bacteria were collected, and the cell pellet was resuspended in lysis buffer (BR0005, ACE, China) and broken by ultrasound. As primary antibodies, polyclonal anti-BaeR, anti-H-NS, and anti-GroEL were used for subsequent immunodetection (Fig. S1). Protein concentrations were determined by Bradford assay to dilute samples to equal levels, and GroEL was used as the loading control (35). The samples were mixed in SDS-loading buffer and boiled for 10 minutes. The proteins were separated by SDS-PAGE and transferred onto nitrocellulose membrane (NC) membranes (66485, Pall, USA). Membranes were blocked with 5% skimmed milk in TBST buffer for 2 h, incubated with a primary antibody for 12 h at 4℃, followed by incubation with a secondary HRP-conjugated anti-mouse goat antibody (7076P2, CST, USA) with a 1:5,000 dilution in 5% BSA for 2 h at room temperature. After washing five times with TBST buffer, signals were detected using the ECL kit (P0018FS, Beyotime, Shanghai, China) following the manufacturer’s protocol.

### Construction of AB43 deletion mutants and complemented strains

The deletion mutants of the *h-ns*, *baeR*, and *cas3* genes in AB43 were generated by a recombineering system *A. baumannii* (32). Briefly, the upstream and downstream homology arms of the target fragment were, respectively, amplified from AB43 genomic DNA and purified. The kanamycin cassette fragment with FRT was amplified by PKD4. Three PCR amplicons containing overlapping regions were assembled using overlap extension PCR with specific primers and introduced by electroporation into *A. baumannii* carrying the plasmid pAT04. After being cultured in 4 mL of LB broth containing 2 mM IPTG for 4 h, the bacteria were pelleted, plated on LB agar with 50 µg/mL kanamycin, and incubated overnight at 37°C. All constructed mutants were verified by PCR, with control primers matching sequences in the genes flanking the deleted open reading frame.

### Transformation of the efficiency assay

The transformation of the efficiency assay was carried out as in a previous study (28). The AB43 and deletion mutants were electroporated with 50 ng CR-sp20 M, CR-sp50 M, CR-sp75 M, or untargeted plasmid and then incubated in 1 mL LB broth for 1 h at 37°C with shaking. Next, they were plated on LB agar containing 50 µg/mL kanamycin and incubated overnight at 37°C. The colony-forming units (CFUs) were counted, and the transformation efficiency was quantified as the percentage transformation by the CRISPR-targeted plasmid compared with the untargeted plasmid.

### Plasmid retention assay

The plasmid retention assay was carried out as in a previous study (28). The AB43 and deletion mutants were transformed with CRISPR-targeted plasmid CR-sp20, CR-sp50, or CR-sp75. Single colonies were cultured in LB broth for 5 h at 37°C with shaking. CFUs were counted on LB agar with and without kanamycin. The percentage of plasmid retention was calculated.

### CRISPR-primed adaptation assay

The CRISPR-primed adaptation assay was carried out as in a previous study (28). The AB43 and deletion mutants were transformed with plasmid CR-sp20 M, CR-sp50 M, or CR-sp75 M, as described above. Single colonies were tested for integration of new immunity spacers and were determined by PCR with 2 × Rapid Taq Master Mix (P222, Vazyme, Nanjing, China). The products were separated by 1.5% agarose gel electrophoresis, and band intensities were quantified using ImageJ2 (2.14.0/1.54 f) (28, 36).

### Biofilm formation assay in plates

Biofilm formation assays were performed in 96-well plates by staining with crystal violet. The bacterial cultures were diluted to 5 × 10^8^ CFU/mL and 1:100 into LB broth. The diluted inoculum was added to a 96-well plate. After incubation for 24 h at 37°C, the liquid contents were discarded, and the wells were washed three times with PBS. The biofilms were then stained with 0.4% crystal violet for 10 min. Excess crystal violet was removed by washing three times with PBS. Finally, 200 µL of 95% ethanol was added to each well, and it was measured as optical density with a 570 nm filter (Bio-Tek SYNERGY2, USA).

### Confocal microscopy imaging of the biofilm

Bacteria cultured in the logarithmic phase of growth were diluted to 5 × 10^6^ CFU/mL in LB broth, and the diluted inoculum was added to a 24-well plate with 14 mm glass climbing tablets (801010, Nest, Wuxi, China). After incubation for 24 h at 37°C, the tablets were washed three times with 0.9% NaCl and stained with SYTO9 (FS4005, FUSHENBIO, Shanghai, China) for 15 min in the dark. The biofilms were observed under a laser confocal scanning microscope (Nikon, Japan), and the images were analyzed using Imaris software.

### Light microscopic imaging of the biofilm

The bacteria were diluted to 5 × 10^6^ CFU/mL and added to a 24-well plate with glass climbing tablets, as described above. After incubation for 24 h at 37°C, the tablets were washed three times with PBS and stained with crystal violet for 15 min. After being washed, the samples were observed under a microscope (Nikon 80i, Japan).

### PNAG assay of biofilm

The PNAG assay of biofilm was carried out as in a previous study (37). The bacteria were diluted to 5 × 10^6^ CFU/mL and added to a 96-well plate. After incubation for 24 h at 37°C, the wells were washed three times with PBS and stained with iFluor 488-labeled Wheat Germ Agglutinin (WGA) (I3300, Solarbio, Beijing, China) for 30 min. The fluorescence was recorded using a Tecan Spark 10M microtiter reader (excitation λ of 488 nm; emission λ of 509 nm).

### RNA extraction and qRT-PCR assay

The strains were cultured overnight in LB broth and then diluted at a ratio of 1:100 into 6 mL LB broth. Following an 8 h incubation period, the cells were harvested for total RNA extraction utilizing the RNAprep pure bacteria kit (RC113, Vazyme, Nanjing, China). For reverse transcription, 350 ng of the extracted RNA was employed, following the HiScript III RT SuperMix protocol for qPCR. Subsequently, 1 µL of the cDNA was utilized for RT-qPCR, which was conducted using the QuantStudio 3 PCR system (Thermofisher, USA). The primers were synthesized by Qinke Biotechnology Company (Beijing, China) (Table S1). The internal control 16SrRNA was employed to standardize gene expression levels. The relative gene expression levels were determined using the 2^-ΔΔCT^ method.

### Bacterial adhesion and invasion assay

In the experiment, the A549 cells were seeded in microplates and cultured to 80% confluency. For bacterial adhesion assay, the uninfected cells were washed three times with PBS. Subsequently, an FBS-free medium without penicillin-streptomycin was used. *A. baumannii* was added to the cell plates at a multiplicity of infection (MOI) of 100 and incubated at 37°C in a 5% CO_2_ atmosphere for 3 h. After the incubation period, the cells were again washed three times with PBS and lysed with 0.1% Triton X-100 (ST797, Beyotime, Shanghai, China). The lysate was plated on LB agar after gradient dilution. For bacterial invasion assay, after the incubation period, the cells were washed three times with PBS, added to the medium containing 100 µg/mL of kanamycin, and incubated for another 15 min. Then, the cells were again washed three times with PBS and lysed with 0.1% Triton X-100. The lysate was plated on LB agar after gradient dilution.

### *Galleria mellonella* killing assay

*Galleria mellonella*, weighing around 300 mg each, were utilized in the study. The injections were administered from the penultimate pair of the right hindfoot using a microsyringe, with each larva receiving the bacterial solution containing roughly 1 × 10^6^ CFUs of bacteria. The control group received injections of PBS. Subsequently, the mortality of the *Galleria mellonella* was monitored and recorded every 12 hours.

### *In vivo* infection assay using the lung infection model

The mice were sourced from the Experimental Animal Center of Yangzhou University in Yangzhou, China. Female BALB/c mice aged 6 to 8 weeks were acclimated for a week before the experiment. A total of 60 mice were randomly grouped into 10 groups: AB43 (control), D*h-ns*, D*baeR*, D*h-ns-baeR*, D*cas3*, D*h-ns-cas3*, D*baeR-cas3*, D*h-ns/ph-hs*, D*baeR/pbaeR*, and D*h-ns-baeR/ph-ns-baeR*. Neutropenia was induced in the mice (each dilution infected 6 mice) by administering cyclophosphamide at doses of 150 mg/kg and 100 mg/kg on days 3 and 1 before infection, respectively. Under anesthesia (2.5% tribromoethanol, M2820, Aibei Biotechnology, Nanjing, China), the mice were injected into the trachea with 60 µL of bacterial suspension (3 × 10^8^ CFUs/mouse). The animals of each group were euthanized on 24 h, and the bronchoalveolar lavage fluid (BALF) was collected, and the lungs were dissected under sterile conditions. The mice were euthanized if severe symptoms developed or if weight loss approached 30% of their initial weight and were scored as dead for humane reasons (38). The tissue was homogenized, and serial 10-fold dilutions were performed for CFU counting.

### Statistical analysis

The data were collected from a minimum of three biological individual experiments, excluding DNA pull-down, and were presented as means ± standard deviations. One-way ANOVA was utilized for multiple-group comparisons, while the log-rank test was employed for analyzing the survival curve. In Western blot analysis, ImageJ was used to quantify band intensities, with protein expression levels normalized to GroEL. Statistical analyses were conducted using GraphPad Prism 10.0. A significance level of *P* < 0.05 was considered statistically significant for all analyses, denoted by * for *P* < 0.05, ** for *P* < 0.01, and *** for *P* < 0.001. Conversely, a nonsignificant difference was indicated by “ns.”

## RESULTS

### Identification of the *cas3* promoter and H-NS binding site

**Fig 1:**
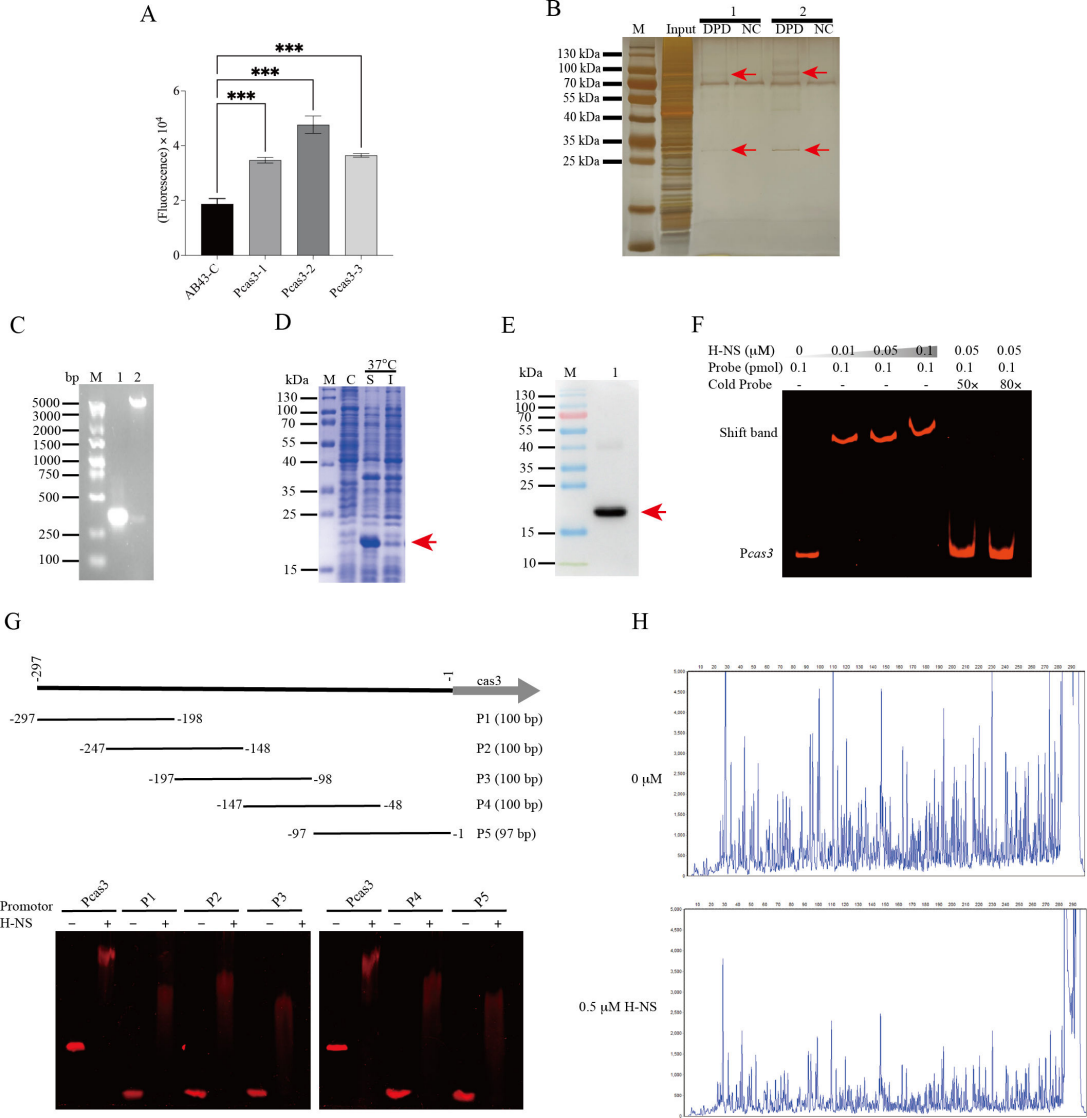
Identification of the *cas3* promoters and the binding site of H-NS activating the *cas3* promoter. (**A**) The reporter plasmids containing empty (AB43-C) and three different lengths of the promoter (163 bp, 297 bp, and 425 bp) were constructed. The activities of putative promoters of *cas3* were measured by fluorescence emission. (**B**) Silver staining analyzed the proteins bound to the *cas3* promoter. 1, 2: two independent experiments. Input: The positive control group is full protein without the probe. DPD: Experimental group is the eluted protein with biotin labeling. NC: negative control group is the eluted protein without biotin labeling. The arrow represents specific bands. (**C**) PCR and double enzyme digestion verification of constructed strains. M: the 5,000 bp DNA marker, 1: PCR products of *h-ns*, 2: double enzyme digestion verification. (**D**) SDS‐PAGE analysis of rH-NS after induction with 1 mM IPTG for 6 h at 37°C. M: the 180 kDa protein marker, C: pET30a without IPTG induction, S: soluble proteins, I: insoluble proteins. (**E**) Western blot analysis of rH-NS with anti-His-tag monoclonal antibody (lane 1). M: the protein marker (**F**) EMSAs for rH-NS binding to the promoter of *cas3*. (**G**) EMSAs of subfragments P1, P2, P3, P4, and P5 with purified rH-NS. (**H**) DNase I footprinting analysis of H-NS binding to the *Cas3* promoter region. The experiments were repeated three times. Error bars show mean ± SD. ***P < 0.001—one-way ANOVA with Dunnett post hoc tests (**A**).

Jutras et al. (39) suggest that biotin-labeled DNA fragments are most effective within the range of 125–425 bp, with the length impacting protein binding. To accurately determine the length of the *cas3* promoter and enhance the reliability of DNA pull-down assays, we constructed reporter plasmids containing three different lengths of the promoter: 163 bp, 297 bp, and 425 bp (Fig. 1A).

Results demonstrated that the 297 bp promoter displayed the highest fluorescence intensity (Fig. 1A). Therefore, all subsequent experiments utilized the 297 bp *cas3* promoter.

To identify regulators of the I-Fb CRISPR-Cas system, we performed DNA pull-down assays using the *cas3* promoter. Silver staining and mass spectrometry revealed H-NS as a direct binding partner (Fig. 1B). MS analysis identified proteins in the upper and the lower band (Table S2). These proteins underwent initial screening, with H-NS selected for further investigation into its regulatory role in Cas3.

To confirm the binding site of H-NS to the *cas3* promoter, we initially engineered a recombinant plasmid (Fig. 1C) and purified rH-NS (Fig. 1D). Subsequently, we detected rH-NS by Western blot (Fig. 1E) and performed EMSAs. EMSAs revealed that the rH-NS bound to the *cas3* promoter in a concentration-dependent manner, completely shifting the DNA fragment (Fig. 1F). This indicates that H-NS binds to the upstream region of the Cas3 operon.

To identify the H-NS binding site in the promoter region of *Cas3*, we performed EMSAs with different, partially overlapping subfragments of the *cas3* promoter (P1, P2, P3, P4, and P5) (Fig. 1G). All five subfragments were shifted after incubation with H-NS.

To precisely define the binding region and to validate the EMSA results, we performed a DNase I footprinting assay. The FAM-labeled *cas3* promoter was mixed with H-NS protein and then digested with DNase I. With the increase in the rH-NS concentration, nearly all regions were protected compared to the control group (Fig. 1H). Upon analyzing the AT content ratio in the *cas3* promoter sequence, a ratio of 60.61% was discovered. This analysis led to the identification of the H-NS-box sequence AT. Consequently, these findings indicate a direct binding of H-NS to the *cas3* promoter, which is AT-rich in nature.

### H-NS outcompetes BaeR in binding the *Cas3* promoter

**Fig 2:**
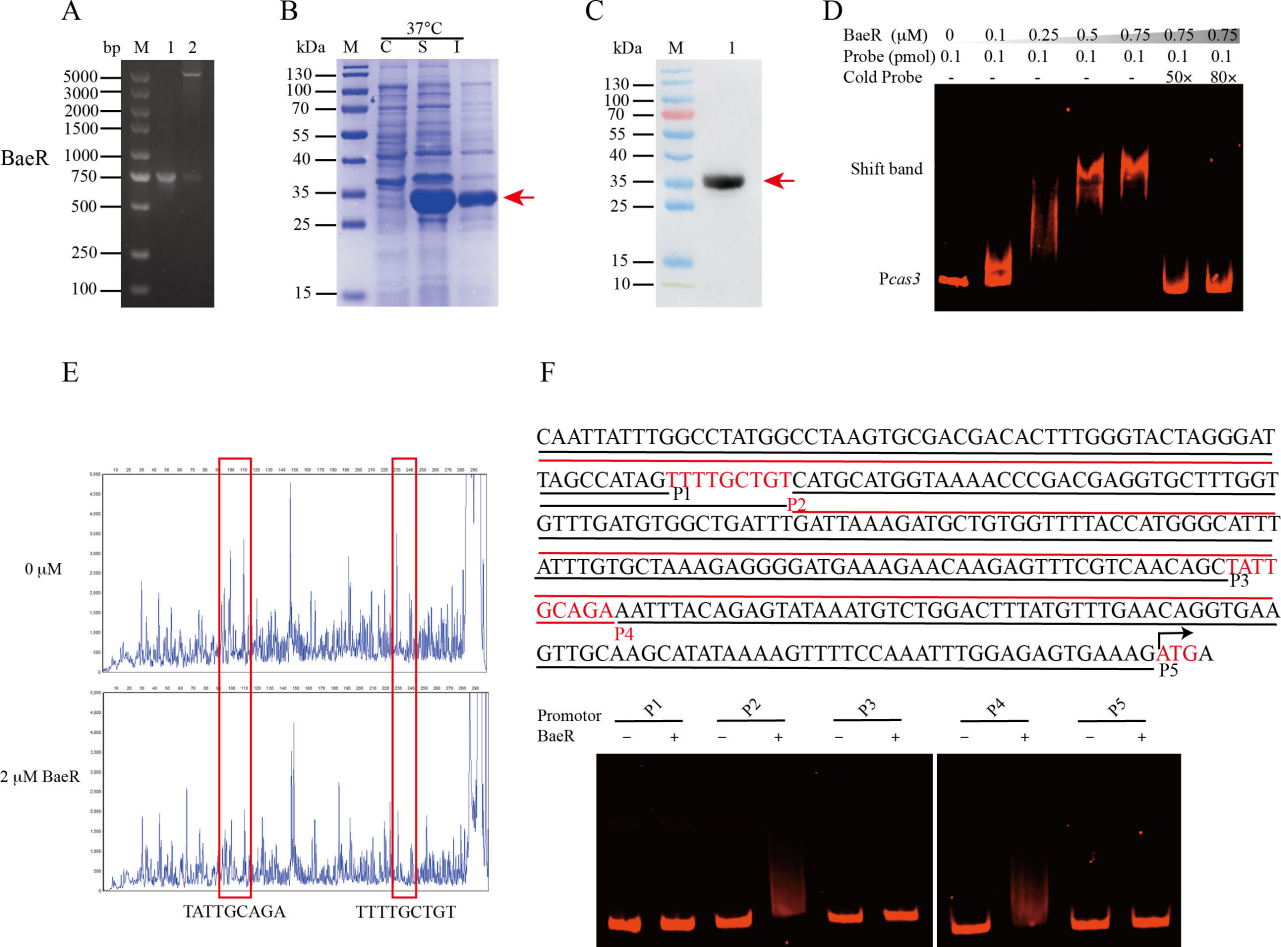
The binding sequences of BaeR activating Cas3 expression. (**A**) PCR and double-enzyme digestion verification of constructed strains. (**B**) SDS‐PAGE analysis of rBaeR after induction with 1 mM IPTG at 37°C for 6 h. M: the 180 kDa protein marker, C: pET30a without IPTG induction, S: soluble proteins, I: insoluble proteins. (**C**) Western blot analysis of rBaeR using an anti‐His mouse monoclonal antibody (lane 1). (**D**) EMSAs for rBaeR binding to the promoter of *cas3*. (**E**) DNase I footprinting analysis of rBaeR binding to the *cas3* promoter region. (**F**) EMSAs of subfragments P1, P2, P3, P4, and P5 with purified rBaeR.

Perez-Rodriguez et al. (29) investigated the BaeSR two-component regulatory system and its role in regulating the I-E CRISPR-Cas systems alongside H-NS in *E. coli*. However, our MS results did not find that BaeR binds to the *cas3* promoter (Table S2). To investigate whether BaeR regulates the Cas3, we constructed recombinant plasmids (Fig. 2A), purified rBaeR (Fig. 2B), and confirmed BaeR by Western blotting (Fig. 2C). Subsequently, we conducted EMSAs, revealing that rBaeR binds to the *cas3* promoter (Fig. 2D). This indicates that BaeR binds to the upstream region of the Cas3 operon. To further identify the BaeR-binding site in the *cas3* promoter region, we performed the DNase I footprinting assay and revealed a protected region spanning positions −241 to −233 relative to the translational start site on the coding strand of the *cas3* promoter with the increasing concentration of rBaeR (Fig. 2E). Similarly, a nearly identical protected region between positions −101 and −93 was observed on the noncoding strand (Fig. 2E). Additionally, we performed EMSAs by overlapping subfragments of the *cas3* promoter (P1, P2, P3, P4, and P5) (Fig. 2F). The P2 and P4 subfragments exhibited a shift after incubation with rBaeR. We identified the BaeR binding box as “TNTTGCNGN.” These findings suggest that the BaeR binds to the *cas3* promoter.

**Fig 3:**
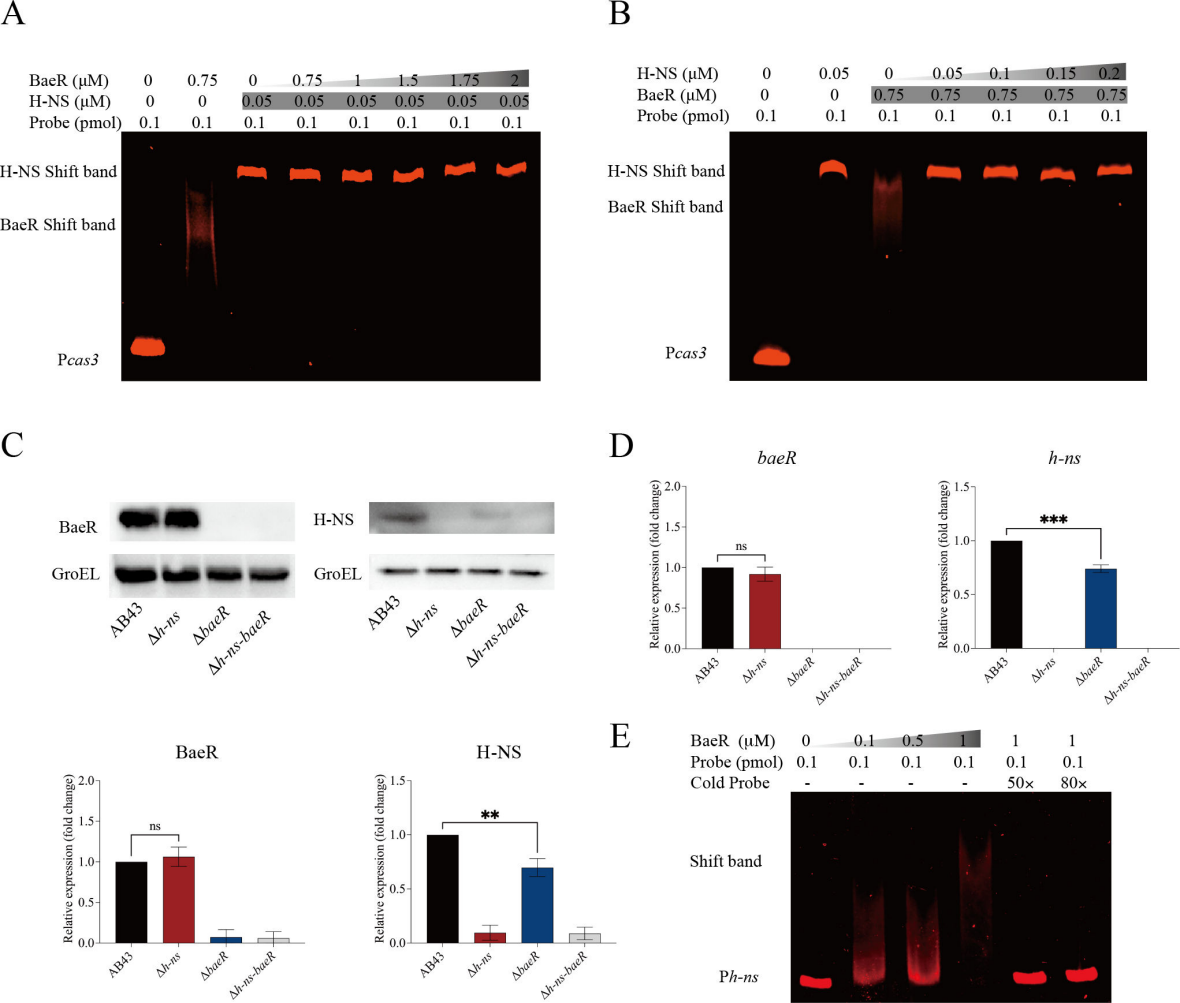
Competitive EMSAs and BaeR increase the H-NS expression. (**A, B**) Competitive EMSAs for binding of rH-NS or rBaeR to the promoters of *cas3*. (**C**) Western blot analysis of H-NS or BaeR expression in mutation strains. (**D**) qRT-PCR analysis of gene *h-ns* or *baeR* expression in mutation strains. (**E**) EMSAs for rBaeR binding to the promoter of *h-ns*. The experiments were repeated three independent times. Data represent mean ± SD. ns *P* > 0.05, **P < 0.01, and ***P < 0.0001—one-way ANOVA with Tukey’s *post hoc* test (**C and D**).

To investigate the competition between BaeR and H-NS in binding to the *cas3* promoter, we performed competitive EMSAs by setting up reactions with both rBaeR and rH-NS added to the *cas3* promoter probe. When maintaining a constant amount of rH-NS but varying concentrations of rBaeR, we observed the migration of the DNA-protein complex resembling that of the H-NS-*cas3* complex (Fig. 3A). Similarly, with increasing concentrations of rH-NS while keeping a constant amount of rBaeR, we observed the formation of the DNA-protein complex characteristic of the H-NS-*cas3* complex (Fig. 3B). These results suggest that H-NS outcompetes BaeR in binding to the promoter probe.

### BaeR promotes the expression of H-NS and represses the Cas3 expression via H-NS

To explore the interplay between BaeR and H-NS, we generated h-ns, baeR mutants, and a double-knockout strain Δ*h-ns-baeR* in AB43, which has a complete I-Fb CRISPR-Cas system. Further investigation showed that the expression of BaeR was unaltered in both Δ*h-ns* and AB43 (Fig. 3C and D). However, compared to AB43, the expression of *h-ns* was significantly decreased in Δ*baeR* (Fig. 3C and D). Additionally, EMSA results demonstrated the binding of rBaeR to the *h-ns* promoter (Fig. 3E). These findings indicated that BaeR promotes the expression of H-NS.

**Fig 4:**
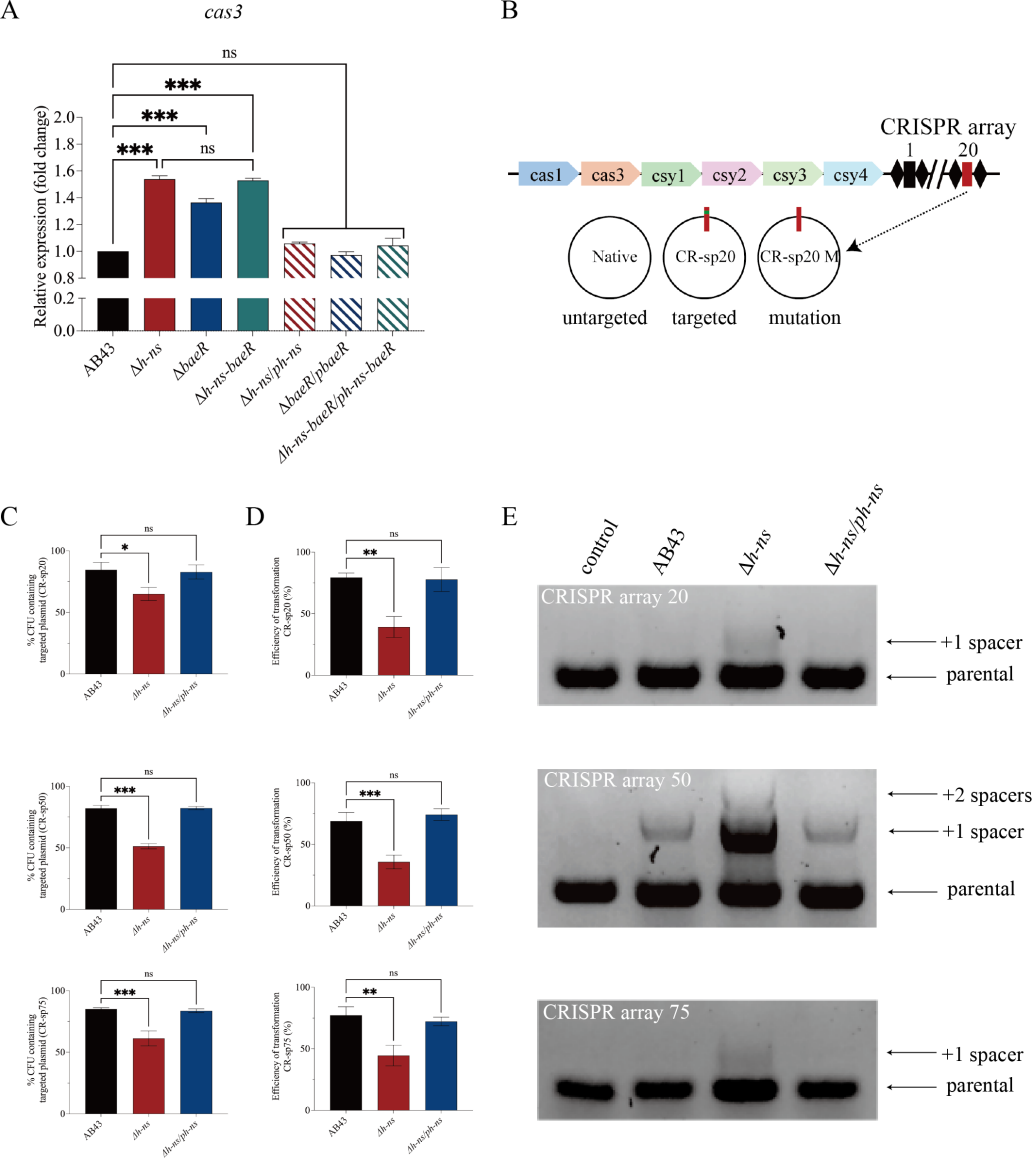
H-NS represses the activity of CRISPR-Cas interference and spacer acquisition. (**A**) The *cas3* expression was measured by qRT-PCR. (**B**) The type I-Fb CRISPR-Cas locus in AB43. A schematic of the experiments utilized a nontargeted plasmid, three CRISPR-targeted plasmids, and three CRISPR-mutation plasmids. (**C**) Retention of the control plasmid and the CRISPR-targeted plasmid in WT and mutants. (**D**) The transformation efficiency of WT and mutants was quantified as the percentage transformation by the CRISPR-targeted plasmid compared with that of the parent vector lacking the targeted sequence. (**E**) Each of the strains harbored the CRISPR-mutation plasmid to promote adaptation. Each adaptation event results in the acquisition of a new spacer and CRISPR repeat. PCR of single colonies analyzed the integration of new CRISPR spacers into the CRISPR locus. Each adaptation event results in the acquisition of a new spacer and CRISPR repeat, which is exhibited by an expansion of the CRISPR locus. Error bars denote the mean ± SD from *n* = 3 replicates. ns *P* > 0.05, *P < 0.05, **P < 0.01, and ***P < 0.001—one-way ANOVA with Tukey’s (**A**) and Dunnett’s (**C and D**) *post hoc* test.

In AB43, the I-Fb CRISPR-Cas system consists of Cas1, Cas3, Csy1-4, and CRISPR arrays. Csy1-4 combine to form a complex with a mature crRNA. Cas3, serving as both a nuclease and a helicase, cleaves DNA tethered by the Csy1-4 complex (31). To investigate how the H-NS regulates the CRISPR-Cas system, we monitored the expression of *cas3*, which encodes the nuclease responsible for cleaving target DNA. Compared to the AB43, D*h-ns* exhibited a significant increase in *cas3* expression (Fig. 4A). Our qRT-PCR analysis revealed a significant increase in *cas3* expression in Δ*baeR* compared to AB43 (Fig. 4A). Compared to Δ*h-ns,* the Δ*baeR* mutant demonstrated a significant reduction in *cas3* expression. In contrast, the double mutant Δ*h-ns-baeR* did not show any significant change in *cas3* expression compared to Δ*h-ns* (Fig. 4A).

The competitive EMSAs demonstrated that H-NS outcompetes BaeR for promoter binding (Fig. 3A and B). While BaeR alone bound the *cas3* promoter, qRT-PCR further revealed that *baeR* deletion significantly reduced *h-ns* expression (Fig. 3D), suggesting BaeR represses the Cas3 expression via H-NS.

### H-NS represses the activity of CRISPR-Cas interference and spacer acquisition

To determine the impact of H-NS on the activity of CRISPR-Cas-mediated interference, we constructed CRISPR-targeted plasmids and evaluated the effect of CRISPR-Cas on eliminating these plasmids (28). The plasmids contained a protospacer targeted by a CRISPR spacer flanked by a protospacer-adjacent motif (PAM) (Fig. 4B). We measured the retention of both CRISPR-untargeted and CRISPR-targeted plasmids in WT AB43 and the Δ*h-ns* after 5 h of growth, as in previous studies (28, 40). While there was no loss of untargeted plasmids in any strains, the targeted plasmids (CR-sp20, 50, and 75) were eliminated by CRISPR-Cas interference in both AB43 and Δ*h-ns*, with the Δ*h-ns* retaining the plasmid significantly less than AB43 (Fig. 4C).

Meanwhile, we examined the influence of H-NS on CRISPR-Cas-mediated elimination of foreign genetic elements by assessing the efficiency of plasmid transformation in WT and mutant strains. The transformation efficiency in Δ*h-ns* was lower than that in AB43, indicating a more effective CRISPR-Cas immune system in the Δ*h-ns* strain (Fig. 4D). These results confirm that H-NS represses the activity of CRISPR-Cas-mediated interference.

Next, to explore the role of H-NS on the activity of CRISPR-Cas-mediated spacer acquisition, we generated a mutation plasmid containing a protospacer with a single base mutation (Fig. 4B). After analyzing individual colonies for expansion of the CRISPR locus by PCR, we observed that CRISPR array 50 had a higher frequency of adaptation compared to CRISPR array 20 and 75 (Fig. 4E). While no new spacers were incorporated into CRISPR arrays 20 and 75 in AB43, the Δ*h-ns* had incorporated one spacer. Moreover, the Δ*h-ns* incorporated an additional spacer into the CRISPR array 50 compared to AB43. These results indicate that H-NS suppresses the activity of CRISPR-Cas-mediated spacer acquisition.

In conclusion, these findings firmly establish that H-NS acts as a negative regulator, inhibiting the expression, interference, and adaptation of the I-Fb CRISPR-Cas system in AB43.

### BaeR represses CRISPR-Cas regulation via H-NS

To evaluate the regulatory role of BaeR in CRISPR-Cas immunity, we conducted a functional analysis encompassing interference activity, spacer acquisition, and the relationships with H-NS.

**Fig 5:**
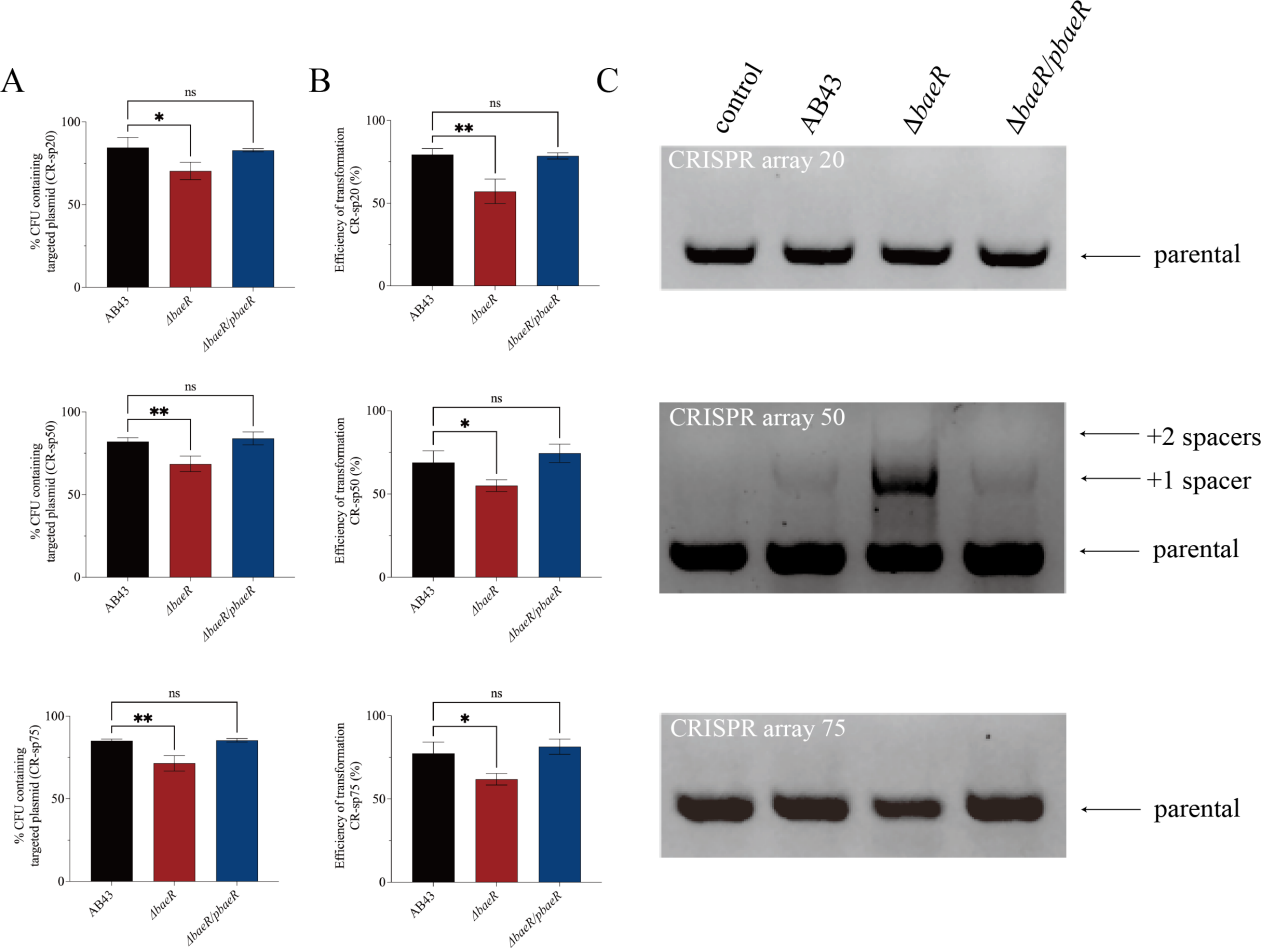
BaeR represses the activity of CRISPR-Cas interference and spacer acquisition. (**A**) Retention of the CRISPR-targeted plasmid. (**B**) Transformation efficiency of CRISPR-targeted plasmids. (**C**) Acquisition of new spacer sequences analyzed by PCR. Error bars represent SD from *n* = 3 replicates. ns *P* > 0.05, *P < 0.05, **P < 0.01, and ***P < 0.001—one-way ANOVA with Dunnett’s (**A and B**) *post hoc* test.

First, we evaluated the retention of CRISPR-targeted plasmids to assess the activity of CRISPR-Cas-mediated interference. The plasmid retention assays revealed that Δ*baeR* exhibited significantly lower retention of the targeted plasmids compared to AB43 (Fig. 5A), suggesting compromised interference efficiency. Meanwhile, the plasmid transformation efficiency assays showed that Δ*baeR* was lower than that in AB43, indicating a more effective CRISPR-Cas immune system in the ΔbaeR strain (Fig. 5B). These results confirm that BaeR represses the activity of CRISPR-Cas targeting activity.

Intriguingly, spacer acquisition analysis demonstrated BaeR’s dual regulatory effects: While CRISPR arrays 20 and 75 showed no new spacer integration in either AB43 or Δ*baeR* (Fig. 5C), array 50 acquired an additional spacer in Δ*baeR*. This suggests BaeR may exert array-specific suppression of spacer integration.

To confirm whether BaeR directly inhibits the activity of CRISPR-Cas-mediated interference of Cas3, we observed that Δ*baeR* exhibited enhanced CR-sp50 plasmid transformation efficiency and plasmid compared to Δ*h-ns*, while CR-sp20 and 75 showed no significant alterations (Fig. S2A and B). Notably, the Δ*h-ns-baeR* double mutant exhibited restoration of plasmid retention levels to Δ*h-ns* baseline (Fig. S2A), indicating that H-NS acts downstream of BaeR in mediating CRISPR interference suppression. Additionally, in Δ*baeR*, there was no insertion of a new fragment in CRISPR arrays 20 and 75, whereas a new fragment was inserted in both Δ*h-ns* and Δ*h-ns-baeR* (Fig. S2C). This aligns with previous studies showing H-NS outcompetes BaeR for the *cas3* promoter, and *baeR* deletion (Δ*baeR*) significantly reduced H-NS expression (Fig. 3E). In summary, BaeR may regulate the immunity of CRISPR-Cas via H-NS.

### BaeR and H-NS modulate biofilm formation and virulence via CRISPR-Cas

**Fig 6:**
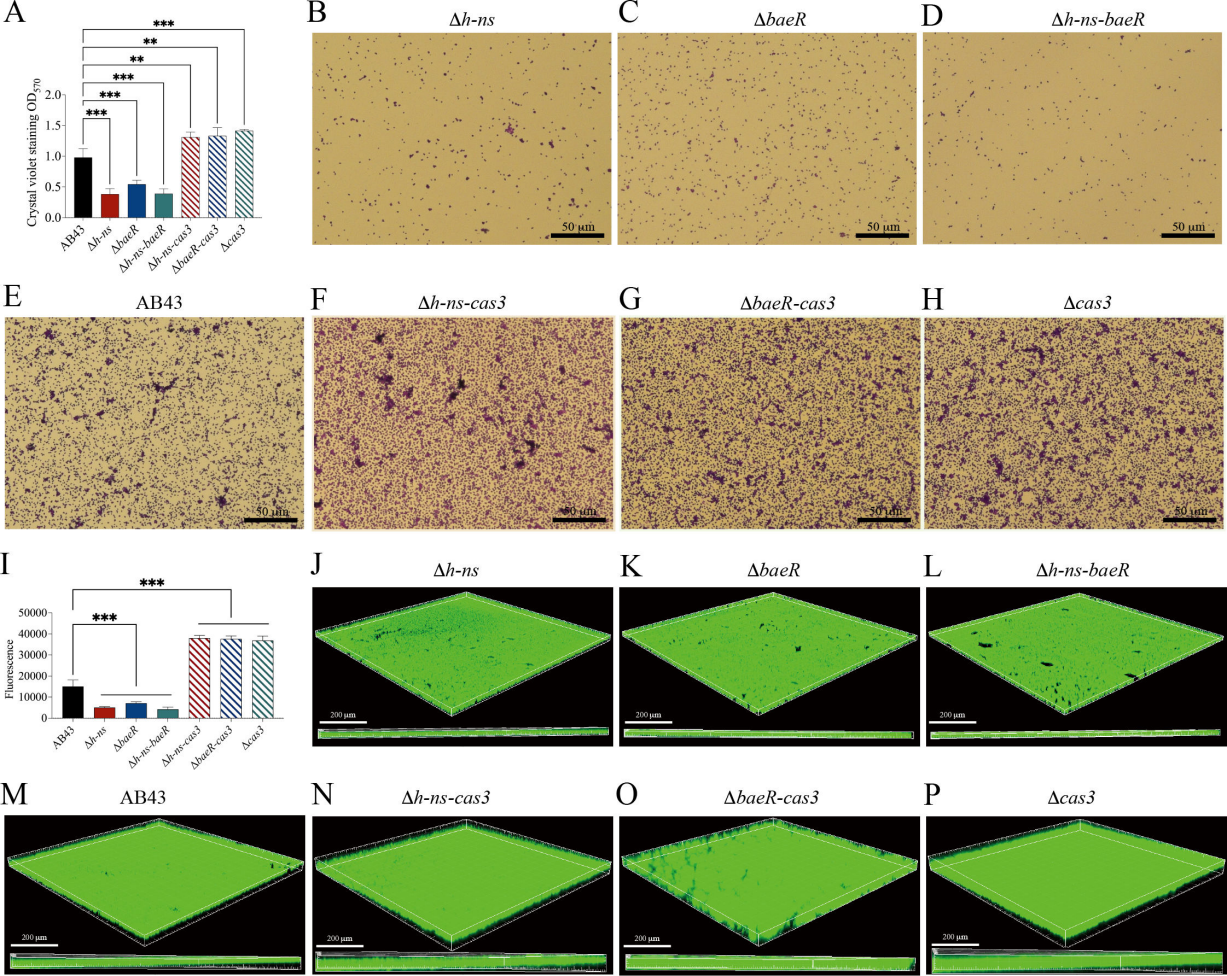
The expression of extracellular matrix components and biofilm formation. (**A**) Measurement of biofilm biomass by crystal violet staining. Data are representative of three independent experiments; bar graphs show mean ± SD. ***P < 0.001—one-way ANOVA with Dunnett’s *post hoc* test. (**B through H**) Light microscopic images of biofilms formed in mutation strains. (**I**) Measurement of extracellular matrix components. Poly N-acetyl glucosamine (PNAG) is a known extracellular matrix component of the hydrophobic biofilm of *A. baumannii*, and the lectin wheat germ agglutinin (WGA) binds selectively to PNAG. Error bars represent SD from *n* = 3 replicates. ***P < 0.001—one-way ANOVA with Dunnett’s *post hoc* test. (**J through P**) Confocal laser microscopic images of biofilms formed by mutation strains on the surface of a 24-well chamber glass slide after 24 h.

**Fig 7:**
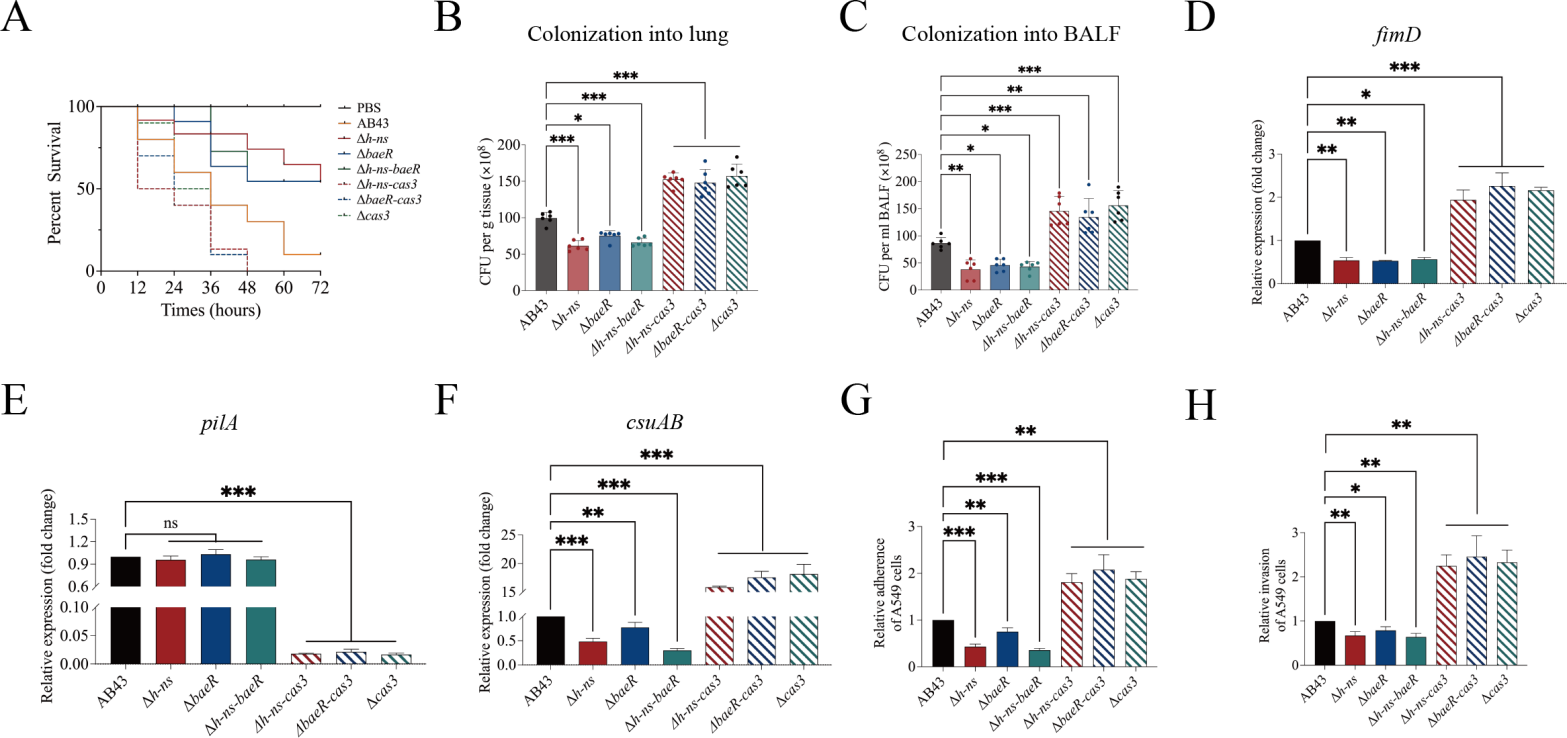
Evaluation of virulence and expression of Csu pili. (**A**) The survival of *Galleria mellonella* (*n* = 10) infected with AB43 and mutants. Survival analyses were performed using Kaplan-Meier survival curves. (**B, C**) The colonization of bacteria into the lungs or BALF of mice was sacrificed after 24 h of intranasal infection and measured by CFU counting of bacterial colonies on LB agar plates. Each experiment was performed with 6 mice. The qRT-PCR analysis of gene *fimD* (**D**)*, csuAB* (**E**), and *pilA* (**F**) expressions. (**G, H**) Adherence and invasiveness of AB43 and mutants to epithelial cells A549. The experiments were repeated three independent times. Error bars show mean ± SD. *P < 0.05, **P < 0.01, and ***P < 0.001—one-way ANOVA with Dunnett’s *post hoc* test.

In addition to being an essential part of the prokaryotic immune system that prevents viral infection, the CRISPR-Cas systems also have various roles in physiology, such as boosting bacterial virulence and countering antibiotic resistance (7, 8, 31, 41). To investigate the regulatory role of the I-Fb CRISPR-Cas system in bacterial virulence, we first analyzed the biofilm-forming abilities and virulence of the Δ*cas3* mutant. Compared to wild-type AB43, the Δ*cas3* strain exhibited significantly enhanced biofilm formation (Fig. 6A through H), accompanied by elevated production of PNAG (Fig. 6I through P), a key extracellular matrix component. This phenotypic shift was further correlated with increased virulence in two infection models. In the *Galleria mellonella* infection model, those infected with Δ*cas3* showed reduced survival rates compared to those infected with AB43 (Fig. 7A). Similarly, in a mouse tracheal intubation model, the Δ*cas3* demonstrated significantly higher bacterial colonization in lung tissues (Fig. 7B) and bronchoalveolar lavage fluid (BALF) (Fig. 7C). These results collectively establish that Cas3 suppresses biofilm formation and virulence in *A. baumannii*.

To dissect the regulatory interplay between BaeR, H-NS, and the CRISPR-Cas system, we detected the biofilm formation abilities of Δ*h-ns* and Δ*baeR*. Both mutants exhibited diminished biofilm formation relative to AB43 (Fig. 6), and this phenotype was reversed upon complementation in the Δ*h-ns/ph-ns* and Δ*baeR/pbaeR* strain (Fig. S3). To determine whether H-NS and BaeR act directly on biofilm regulation or indirectly via Cas3 in AB43, we constructed Δ*h-ns-cas3* and Δ*baeR-cas3* double mutants. Strikingly, the biofilm-forming capacity of these double mutants was indistinguishable from that of the Δ*cas3* single mutant, yet all three mutants (Δ*cas3*, Δ*h-ns-cas3*, and Δ*baeR-cas3*) produced significantly more biofilms than AB43 (Fig. 6). These findings suggest that H-NS and BaeR regulate biofilm formation through Cas3, which is the primary inhibitor of this process.

Consistent with biofilm formation, PNAG quantification revealed that Δ*h-ns* and Δ*baeR* single mutants produced significantly less extracellular matrix component than AB43 (Fig. 6I), and this phenotype was reversed upon complementation in the Δ*h-ns/ph-ns* and Δ*baeR/pbaeR* strain (Fig. S3F). In contrast, Δ*h-ns-cas3* and Δ*baeR-cas3* showed PNAG levels comparable to those of the Δ*cas3* strain (*P* > 0.05), with all three mutants exceeding AB43 in PNAG production (Fig. 6I). These data reinforce that Cas3 is the central suppressor of biofilm-associated extracellular matrix component synthesis, while H-NS and BaeR exert their regulatory effects upstream, dependent on Cas3 functionality.

In the *Galleria mellonella* infection model, Δ*h-ns* and Δ*baeR* caused significantly lower mortality compared to AB43, whereas Δ*cas3*, Δ*h-ns-cas3*, and Δ*baeR-cas3* strains exhibited similarly elevated lethality (Fig. 7A). Parallel results were observed in the mouse model: Δ*h-ns* and Δ*baeR* mutants showed reduced bacterial colonization in lung tissues and BALF, while double mutants mirrored the hypervirulent phenotype of Δ*cas3* (Fig. 7B and C). These findings conclusively link H-NS and BaeR to a Cas3-dependent pathway governing virulence.

### BaeR and H-NS regulate pili via CRISPR-Cas

The biofilm of *A. baumannii* enhances its colonization and survival on both biological and abiotic surfaces, thereby exerting its virulence (37). Bacterial pili not only play a role in biofilm formation but are also considered essential virulence factors for host cell adhesion (42).

To explore whether the CRISPR-Cas system modulates biofilm formation through pili, we first analyzed pili-associated gene expression in the Δ*cas3*. Quantitative RT-PCR revealed significant upregulation of *fimD* and *csuAB* alongside downregulation of *pilA* (Fig. 7D through F) in Δ*cas3* compared to AB43. Consistent with these changes, Δ*cas3* exhibited enhanced adhesion (Fig. 7G) and invasion (Fig. 7H) in A549 cells, suggesting that Cas3 suppresses biofilm-associated virulence by repressing specific pili systems.

To dissect the functional contributions of two major pilus types—Csu and type IV pili—we generated Δ*pilA* (type IV pili) and Δ*csuAB* (Csu pili) mutants. While Δ*pilA* showed no significant change in adhesion compared to AB43 (*P* > 0.05), Δ*csuAB* displayed a reduction in adhesion (Fig. S4). These results establish Csu pili as the primary mediator of biofilm-related adhesion and virulence in *A. baumannii*.

To determine whether the BaeR and H-NS influence pili activity via CRISPR-Cas, we assessed adhesion ability in Δ*baeR*, Δ*h-ns*, Δ*h-ns-cas3*, and Δ*baeR-cas3* mutants. Both Δ*baeR* and Δ*h-ns* exhibited reduced adhesion compared to AB43, and this phenotype was reversed upon complementation in the Δ*h-ns/ph-ns* and Δ*baeR/pbaeR* strain (Fig. S4). Notably, Δ*h-ns-cas3* and Δ*baeR-cas3* double mutants showed adhesion levels indistinguishable from Δ*cas3* (*P* > 0.05), but all three strains (Δ*cas3*, Δ*h-ns-cas3,* and Δ*baeR-cas3*) exceeded AB43 in adhesion (Fig. 7G). QRT-PCR results demonstrated that *fimD* and *csuAB* expressions were significantly reduced in Δ*baeR* and Δ*h-ns* (Fig. 7F through H), while that of *pilA* remained unaffected (*P* > 0.05). These findings indicate that BaeR and H-NS regulate Csu pili via Cas3, rather than directly controlling pilus genes.

## DISCUSSION

The CRISPR-Cas system, a prokaryotic adaptive immune mechanism, selectively degrades foreign genetic elements and modulates virulence factor expression, thereby influencing host-pathogen interactions (6). Current studies have revealed that approximately 39% of sequenced bacterial and 88% of archaeal genomes harbor CRISPR-Cas systems (43). Notably, the clinical carriage rate of CRISPR-Cas systems in *A. baumanni*i reaches 46.12% (31), significantly higher than the average level reported in the bacterial kingdom, suggesting that this system may play an especially important role in this pathogen. The CRISPR-Cas system in *A. baumannii* is primarily classified into types I-Fa and I-Fb (44). The I-Fa CRISPR-Cas system exhibits a potential virulence-enhancing effect (45), whereas the I-Fb subtype demonstrates an inhibitory function (31). This observed “functional dichotomy” within the same type of system strongly suggests that CRISPR-Cas is not merely an independent immune module in *A. baumannii* but rather a critical factor deeply embedded in its global regulatory network, capable of directly influencing bacterial pathogenicity.

However, the core transcriptional regulatory mechanisms mediating the crosstalk between CRISPR-Cas immune function and virulence output remain largely unknown in *A. baumannii*. Although studies in other pathogens, such as *Pseudomonas aeruginosa* and *Staphylococcus aureus*, have identified specific transcription factors that integrate environmental signals to precisely regulate the expression of CRISPR-cas gene clusters (28, 40, 46, 47), whether such a regulatory network exists in *A. baumannii* and how it operates has yet to be elucidated.

Here, we elucidate a hierarchical regulatory axis in the *A. baumannii* I-Fb CRISPR-Cas system, where BaeR and H-NS coordinately govern immune defense and virulence. We demonstrate that the H-NS directly binds AT-rich regions within the *cas3* promoter, effectively suppressing CRISPR-Cas interference and adaptive immunity. H-NS, as a DNA-binding factor, typically plays a role in coordinating the transcriptional expression of host-related functions in many bacteria (48). As a conserved DNA-bridging protein, H-NS acts as a transcriptional silencer by stabilizing AT-rich DNA structures (49), consistent with its CRISPR-inhibitory roles in *Escherichia coli* (25, 26), *Klebsiella pneumoniae* (50), and *Salmonella enterica* (51). Notably, in *A. baumannii* I-Fa systems, Kim et al. (52) previously identified H-NS as a Cas3 repressor, which was corroborated in our I-Fb system.

Intriguingly, the two-component system BaeR exhibits atypical regulatory behavior. While DNA pull-down assays failed to detect direct BaeR-*cas3* promoter interactions, EMSA revealed partial binding of rBaeR to the *cas3* promoter. Paradoxically, CRISPR activity decreased in Δ*baeR*, yet Δ*h-ns-baeR* double knockouts showed no additive suppression. This suggests indirect regulation, further supported by BaeR-mediated upregulation of H-NS expression and direct rBaeR binding to the *h-ns* promoter. This hierarchical cascade contrasts sharply with *E. coli* (29), where BaeSR directly activates *cas* genes, underscoring evolutionary divergence in gram-negative CRISPR-Cas regulation.

In addition to its immune function against invasive genetic material, the CRISPR-Cas system is closely associated with bacterial resistance and virulence (7, 8, 53, 54). We uncover a virulence-modulatory role for the I-Fb CRISPR-Cas system. The Δ*cas3* mutants exhibited enhanced biofilm formation, increased extracellular matrix component production, and impaired A549 cell adhesion. *A. baumannii* predominantly possesses IV-type pili (5–140 nm) and Csu pili (140–1,000 nm) (55). We constructed Δ*pilA* and Δ*csuAB* strains and confirmed that Cas3 selectively represses the Csu pili while leaving type IV pili (pilA) unaffected, which is a critical colonization factor as reported by Ahmad et al. (37).

Our results suggest that the deletion of *cas3* leads to excessive accumulation of biofilm and PNAG and increased expression of pili, which ultimately enhances the colonization ability and pathogenicity of bacteria in the host. Notably, we found that the biofilm and virulence functions of the bacteria decreased in Δ*baeR* and Δ*h-ns* in CRISPR-positive AB43, as well as the pili and adhesion functions. Our findings contrast with those of studies in CRISPR-negative strain ATCC 17978, where H-NS deletion increased adhesion and virulence (Eijkelkamp et al.) (56) and BaeR mutation enhanced pili-mediated biofilm formation (Liu et al.) (57). This divergence implies CRISPR-dependent regulatory rewiring in strain AB43: BaeR and H-NS may suppress Cas3 to derepress virulence factors, whereas CRISPR-less strains employ alternative pathways. Further studies showed that the Δ*h-ns-cas3* and Δ*baeR-cas3* double-knockout strains exhibited similar virulence phenotypes to the Δ*cas3* single-knockout strains (*P* > 0.05), suggesting that they formed a regulatory cascade through the CRISPR-Cas system. Δ*h-ns-cas3* and Δ*baeR-cas3* strains phenocopied Δ*cas3* in virulence assays (*P* > 0.05), establishing a linear regulatory hierarchy: BaeR/H-NS → Cas3 → biofilm/EPS → Csu pili → virulence.

In the study, H-NS serves as the primary regulator of CRISPR-Cas-mediated immune defense and virulence modulation, while BaeR exhibits only ancillary regulatory input. Notably, the subtle modulatory role of BaeR observed here may reflect condition-dependent functionality. The two-component system BaeSR is a key hub for bacterial environmental responses, and the weak effects of BaeR may only be amplified under specific conditions (such as sub-inhibitory antibiotic exposure). We need to further explore the characteristics of the stress response.

In summary, our study provides the first evidence that *A. baumannii* employs the BaeR and H-NS regulators to suppress the immune function of the type I-Fb CRISPR system and modulate bacterial virulence, thereby maintaining cellular homeostasis. Based on these findings, the identification of CRISPR-associated transcription factors specific to *A. baumannii* could pave the way for a “precision interference” therapeutic approach. For instance, small-molecule agonists could be designed to specifically enhance I-Fb system activity to attenuate virulence, or inhibitors could be used to dampen I-Fa system function. Such a strategy targeting regulatory pathways may help mitigate the evolution of resistance driven by conventional antibiotics. However, a major challenge for clinical translation lies in the high heterogeneity of *A. baumannii* clinical isolates. Thus, any CRISPR-Cas-based therapeutic strategy must be coupled with rapid molecular diagnostics to distinguish between CRISPR-negative, I-Fa, and I-Fb strains, enabling truly precision medicine.

## SUPPLEMENTAL MATERIAL

Fig S1 msystems.01067-25-s0001.tif Humoral immune responses in immunized mice.

Fig. S2 msystems.01067-25-s0002.tif Activity of CRISPR-Cas interference and spacer acquisition.

Fig. S3 msystems.01067-25-s0003.tif Biofilm formation and measurement of extracellular matrix component PNAG.

Fig. S4 msystems.01067-25-s0004.tif Evaluation of virulence, invasive, and adherence.

Legends msystems.01067-25-s0005.docx Supplemental figure legends.

Table S1 msystems.01067-25-s0006.docx Primers.

Table S2 msystems.01067-25-s0007.docx Identification of proteins to the *cas3* promoter region by MS.

ASM does not own the copyrights to Supplemental Material that may be linked to, or accessed through, an article. The authors have granted ASM a non-exclusive, world-wide license to publish the Supplemental Material files. Please contact the corresponding author directly for reuse.
