# Supplementary figures and images for: BaeR and H-NS control CRISPR-Cas-mediated immunity and virulence in Acinetobacter baumannii
Source: mSystems. 2025 Oct 31;10(11):e01067-25. doi: 10.1128/msystems.01067-25 (PMC12625773; doi:10.1128/msystems.01067-25)

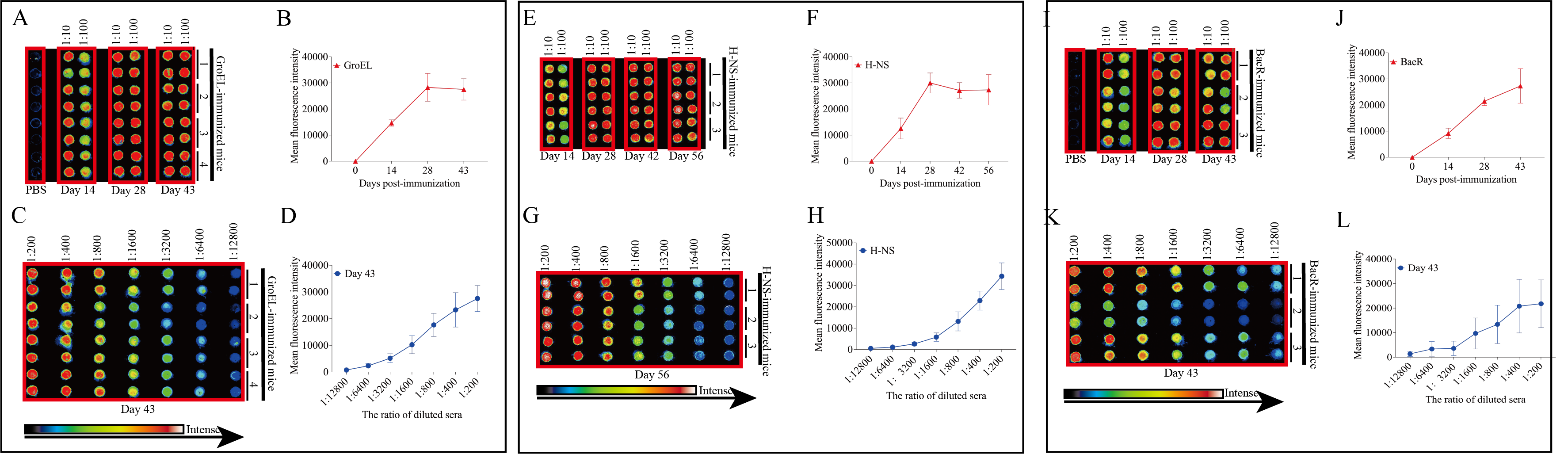

Supplement: Fig S1 — Humoral immune responses in immunized mice. [file msystems.01067-25-s0001.tif]

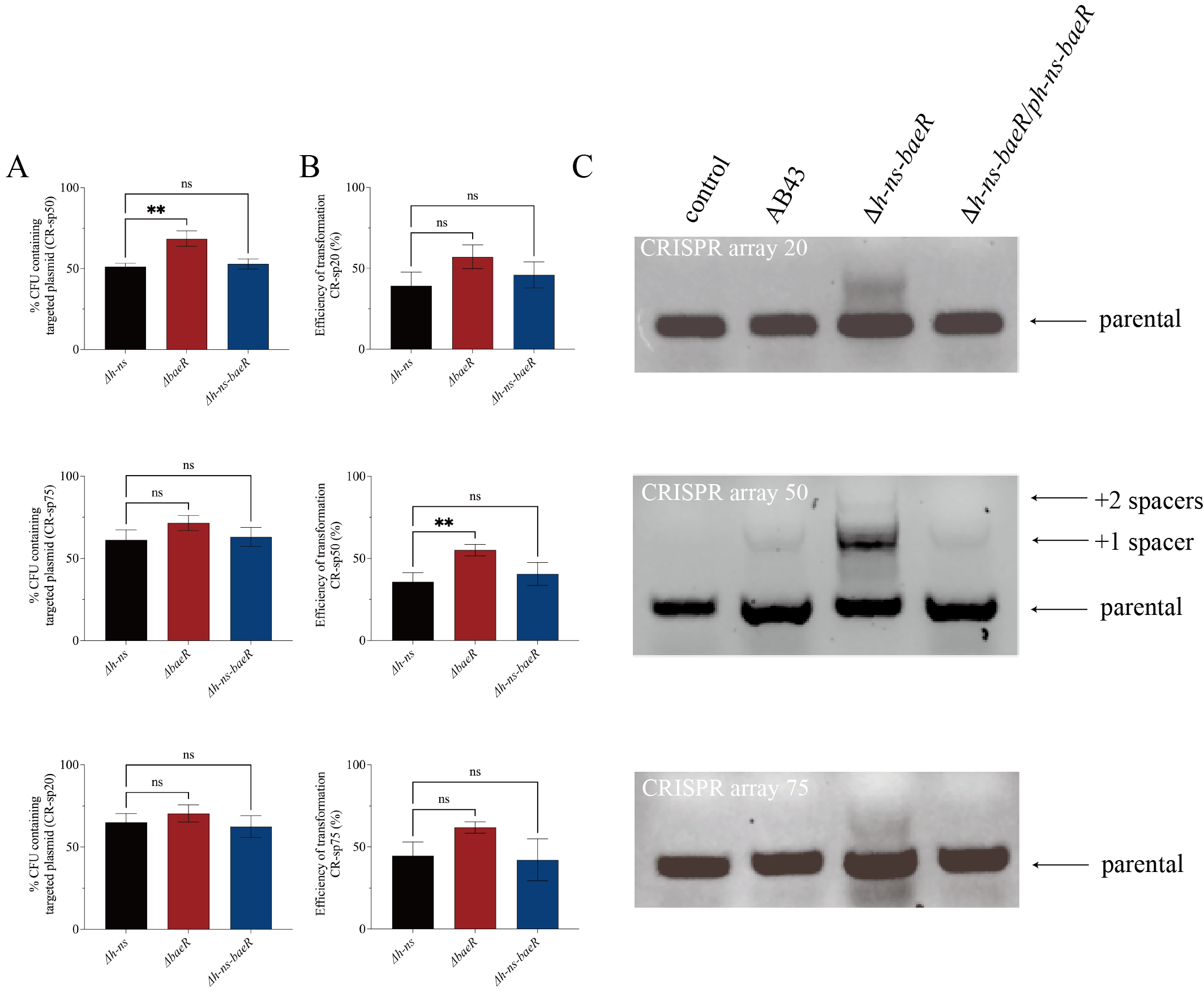

Supplement: Fig. S2 — Activity of CRISPR-Cas interference and spacer acquisition. [file msystems.01067-25-s0002.tif]

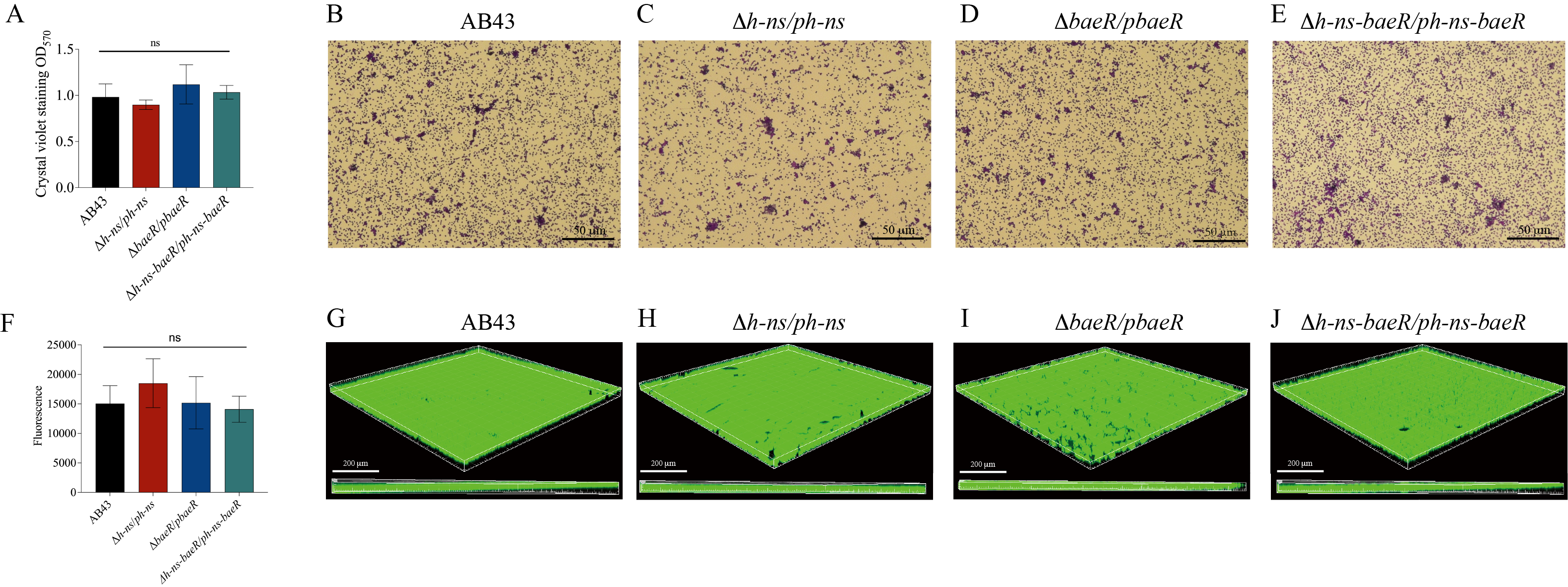

Supplement: Fig. S3 — Biofilm formation and measurement of extracellular matrix component PNAG. [file msystems.01067-25-s0003.tif]

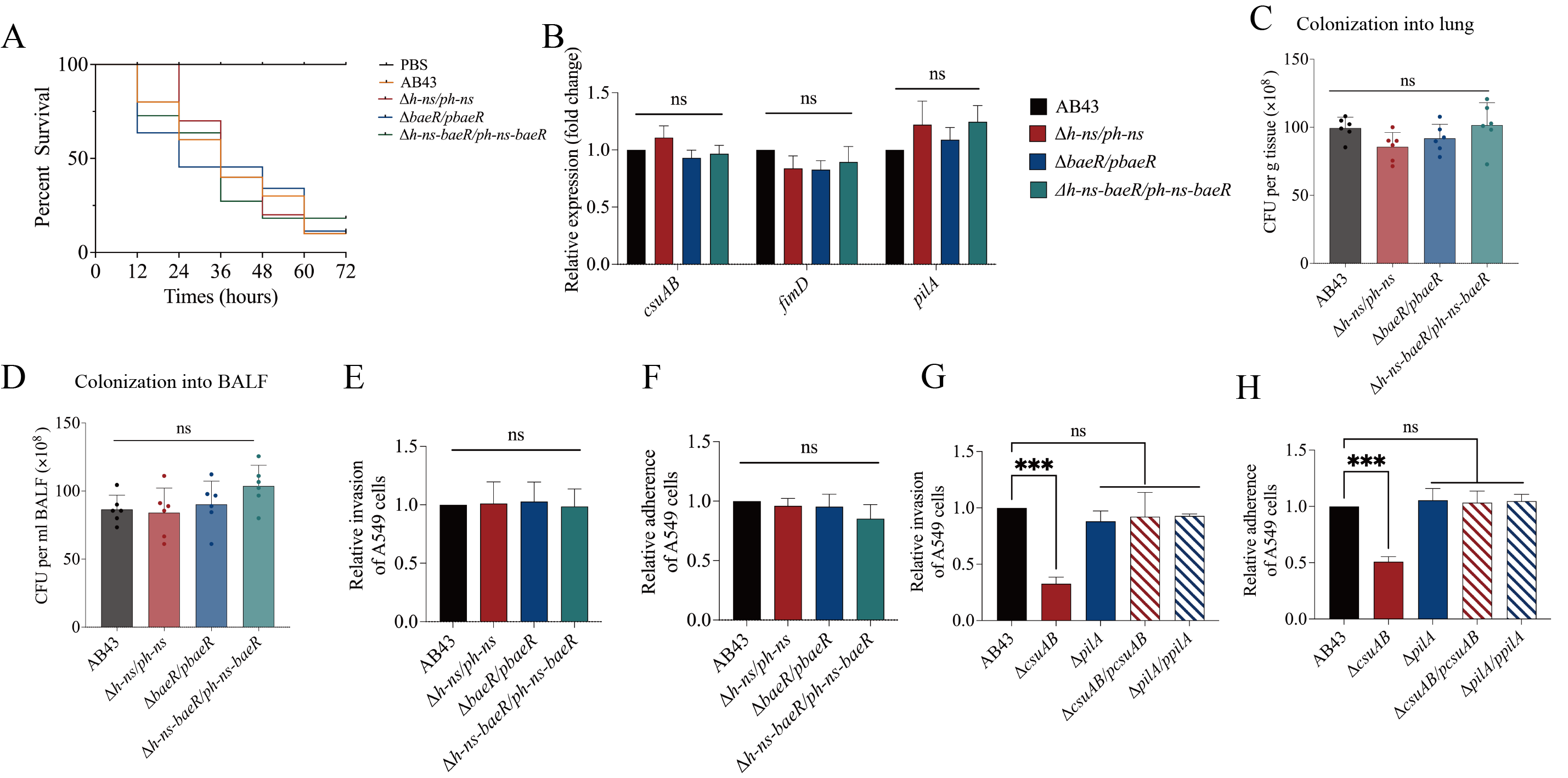

Supplement: Fig. S4 — Evaluation of virulence, invasive, and adherence. [file msystems.01067-25-s0004.tif]
