# Supplementary material for: BaeR and H-NS control CRISPR-Cas-mediated immunity and virulence in Acinetobacter baumannii
Source: mSystems. 2025 Oct 31;10(11):e01067-25. doi: 10.1128/msystems.01067-25 (PMC12625773; doi:10.1128/msystems.01067-25)
Supplement: Legends — Supplemental figure legends. [file msystems.01067-25-s0005.docx]

Figure S1: Humoral immune responses in immunized mice. To evaluate humoral immune responses, female BALB/c mice (6 weeks old) were procured from the Experimental Animal Center of Yangzhou University (Yangzhou, China). The mice were subcutaneously immunized with an emulsion containing 50 μg of protein formulated with Freund’s complete adjuvant (Sigma, catalog no. F5881) as the primary immunization. Booster immunizations were administered on days 14 and 28 post-primary immunization using the same dose of antigen emulsified in Incomplete Freund’s adjuvant (Sigma-Aldrich, catalog no. F5506). Blood samples were collected on days 0, 14, 28, 42, and 70, and serum was separated and stored at −80 °C for subsequent analysis. (A, E, I) Levels of total IgG in mouse serum against protein were measured by protein arrays on days post-immunization. A false color image of an example array shows signal intensity; a white or red spot is more intense than the blue ones. The number at the top of the picture represents different mice. (B, F, J) Quantification of the relative spot intensities of duplicates. Higher concentrations of antibody in the serum produce stronger mean fluorescence intensity. (C, G, K) Serum from immunized mice was arrayed in duplicate in a series of two‐fold dilutions. (D, H, L) Linearity and replicability of this data are shown in the graph.

Figure S2: The activity of CRISPR-Cas interference and spacer acquisition. (A) Retention of the CRISPR-targeted plasmid. (B) Transformation efficiency of CRISPR-targeted plasmids. (C) Acquisition of new spacer sequences analyzed by PCR. Error bars represent SD from n = 3 replicates. ns p>0.05, *p<0.05, **p<0.01 — One-Way ANOVA with Tukey (A & B) post-hoc test.

Figure S3: Biofilm formation and Measurement of extracellular matrix components PNAG. (A) Measurement of biofilm biomass by crystal violet staining. (B-E) Light microscopic images of biofilms formed. (F) Measurement of extracellular matrix components PNAG. (G-J) Confocal laser microscopic images of biofilms formed by strains on the surface of a 24-well chamber glass slide after 24 h. Data are representative of three independent experiments; bar graphs show mean ± SD.

Figure S4: Evaluation of virulence, invasive, and adherence. (A) The survival of Galleria mellonella (n = 10) infected with AB43 and complement strains. Survival analyses were performed using Kaplan-Meier survival curves. (B) The qRT-PCR analysis of gene *fimD, csuAB* and *pilA* expression. (C, D) The colonization of bacteria into the lungs or BALF of mice was sacrificed after 24 h of intranasal infection and measured by CFU counting of bacterial colonies on LB agar plates. Each experiment was performed with 6 mice. (E-H) Invasive and adherence of AB43 and mutants to epithelial cells A549. Error bars show mean ± SD. ns p>0.05, ***p<0.001 — two-way ANOVA with Tukey's post hoc (B) and One-Way ANOVA with Dunnet post-hoc test (C-F).
