# Supplementary material for: BaeR and H-NS control CRISPR-Cas-mediated immunity and virulence in Acinetobacter baumannii
Source: mSystems. 2025 Oct 31;10(11):e01067-25. doi: 10.1128/msystems.01067-25 (PMC12625773; doi:10.1128/msystems.01067-25)
Supplement: Table S1 — Primers. [file msystems.01067-25-s0006.docx]

Table S1: List of primers used in this study

| Name | Sequene (5’ − 3’) | Length (bp) | Notes |
| --- | --- | --- | --- |
| ABori-F1 | cggactagtgatcgtagaaatatctatgattatcttgaag | 1337 | F for the origin of replication cloning to pBR322-Tac (SpeI) |
| ABori-R1 | cggactagtggattttaacattttgcgttgttcc |  | R for the origin of replication cloning to pBR322-Tac (SpeI) |
| ABori-F2 | atcgatcggatcgtagaaatatctatgattatcttgaag | 1337 | F for the origin of replication cloning to pBBR1MCS-Tac-EGFP (PvuI) |
| ABori-F2 | cggactagtggattttaacattttgcgttgttcc |  | F for the origin of replication cloning to pBBR1MCS-Tac-EGFP (PvuI) |
| pET30a-groEL-F | gctgatatcggatccgaattctcagctaaagacgtaaaatttgg | 1635 | F for *04125* cloning to pET30a (EcoRI) |
| pET30a-groEL-R | gtggtggtggtggtgctcgagcatcattccgcccatacc |  | R for *04125* cloning to pET30a (XhoI) |
| pET30a-BaeR-F | gccatggctgatatcggatccaaacatattatgttggttgagg | 687 | F for *BaeR* cloning to pET30a (BamHI) |
| pET30a-BaeR-R | gtggtggtggtggtgctcgagttcttctggatattcgaagc |  | R for *BaeR* cloning to pET30a (XhoI) |
| pET30a-H-NS-F | gctgatatcggatccgaattcaaaccggatattagtgaa | 327 | F for *H-NS* cloning to pET30a (EcoRI) |
| pET30a-H-NS-R | gtggtggtggtggtgctcgaggattaagaaatcttcaagttt |  | R for *H-NS* cloning to pET30a (XhoI) |
| pET30a-Cas3-F | gccatggctgatatcggatccatcgtaacctttatctctc | 3375 | F for *H-NS* cloning to pET30a (BamHI) |
| pET30a-Cas3-R | gtggtggtggtggtgctcgagtttatccaaaactacaagtcc |  | R for *H-NS* cloning to pET30a (XhoI) |
| PCas3-F1 | gtacaagtaatttttgaattctggttttaccatgggcatt | 163 | F for -163 to 0 of Cas3 cloning to pBEAB (EcoRI) |
| PCas3-F2 | gtacaagtaatttttgaattccaattatttggcctatggcc | 297 | F for -297 to 0 of Cas3 cloning to pBEAB (EcoRI) |
| PCas3-F3 | gtacaagtaatttttgaattcttcctaacatgactaaggt | 425 | F for -425 to 0 of Cas3 cloning to pBEAB (EcoRI) |
| PCas3-R | gtcgacggtatcgataagcttctttcactctccaaatttgg |  | R for putative of Cas3 cloning to EGFP-ABori (HindIII) |
| *h-ns*-up-F | ccaagcgattctgcttggcttt | 194 | F for upstream of Δ*H-NS* |
| *h-ns*-up-R | ccaagcgattctgcttggcttt |  | R for upstream of Δ*H-NS* |
| *h-ns*-down-F | ctccagtcttactgacaattc | 224 | F for downstream of Δ*H-NS* |
| *h-ns*-down-R | gaaaaatcagttgggcatg |  | R for downstream of Δ*H-NS* |
| *h-ns*-kan-F | gaattgtcagtaagactggaggtgtaggctggagctgcttc | 1478 | F for Δ*H-NS* kan cassette |
| *h-ns*-kan-R | aaagccaagcagaatcgcttggggtccatatgaatatcctcc |  | R for Δ*H-NS* kan cassette |
| *h-ns*-com-F | ccctttcgtcttcaagaattcggtgaagcagttatggtttg | 675 | F for complementation of Δ*H-NS* (EcoRI) |
| *h-ns*-com-R | taaactaccgcattaaagcttcgcttggctttttaagaggc |  | R for complementation of Δ*H-NS* (HindIII) |
| *baeR*-up-F | ttatcgtgtcgatgattcgc | 141 | F for upstream of Δ*BaeR* |
| *baeR*-up-R | gtcagtacacaacgtaaacc |  | R for upstream of Δ*BaeR* |
| *baeR*-down-F | tatgctgtggataaccacaa | 227 | F for downstream of Δ*BaeR* |
| *baeR-*down-R | taaccatatggcacaacgag |  | R for downstream of Δ*BaeR* |
| *baeR*-kan-F | ggtttacgttgtgtactgacgtgtaggctggagctgcttc | 1518 | F for Δ*BaeR* kan cassette |
| *baeR*-kan-R | ttgtggttatccacagcataccatatgaatatcctccttag |  | R for Δ*BaeR* kan cassette |
| *baeR*-com-F | ccctttcgtcttcaagaattcgtgcttctttattgttagaccg | 1096 | F for complementation of Δ*BaeR* (EcoRI) |
| *baeR*-com-R | taaactaccgcattaaagcttccaacataatatgtttcatgggcc |  | R for complementation of Δ*BaeR* (HindIII) |
| *cas3*-up-F | tgaacaggtgaagttgcaagca | 154 | F for upstream of Δ*Cas3* |
| *cas3*-up-R | tgccatgtgttattaccaatccg |  | R for upstream of Δ*Cas3* |
| *cas3*-down-F | acaacttaccttttccgagca | 153 | F for downstream of Δ*Cas3* |
| *cas3*-down-R | atggcactttcattttcggc |  | R for downstream of Δ*Cas3* |
| *cas3*-kan-F | cggattggtaataacacatggcagtgtaggctggagctgcttc | 1478 | F for Δ*Cas3* kan cassette |
| *cas3*-kan-R | tgctcggaaaaggtaagttgtccatatgaatatcctccttag |  | R for Δ*Cas3* kan cassette |
| *h-ns*-*baeR*-com-F1 | ccctttcgtcttcaagaattcggtgaagcagttatggtttg | 675 | F for complementation of Δ*H-NS-BaeR* (EcoRI) |
| *h-ns*-*baeR*-com-R1 | ggtctaacaataaagaagcactcgcttggctttttaagaggc |  | R for complementation of Δ*H-NS-BaeR* |
| *h-ns*-*baeR*-com-F2 | gtgcttctttattgttagacc | 1096 | F for complementation of Δ*H-NS-BaeR* |
| *h-ns*-*baeR*-com-R2 | taaactaccgcattaaagctttgtggttatccacagcataac |  | R for complementation of Δ*H-NS-BaeR* (HindIII) |
| *csuAB*-up-F | ctttccgagcaattgcatt | 172 | F for upstream of Δ*CsuAB* |
| *csuAB*-up-R | gatttcggcttattggatatc |  | R for upstream of Δ*CsuAB* |
| *csuAB*-down-F | attgagtggcaggtttgc | 132 | F for downstream of Δ*CsuAB* |
| *csuAB*-down-R | agctccaatttcacccgc |  | R for downstream of Δ*CsuAB* |
| *csuAB*-kan-F | gatatccaataagccgaaatcgtgtaggctggagctgcttc | 1517 | F for Δ*CsuAB* kan cassette |
| *csuAB*-kan-R | gcaaacctgccactcaatccatatgaatatcctccttag |  | R for Δ*CsuAB* kan cassette |
| *csuAB*-com-F | ccctttcgtcttcaagaattcagccttagatatctaagccac | 602 | F for complementation of Δ*CsuAB* (EcoRI) |
| *csuAB*-com-R | taaactaccgcattaaagcttgcaaacctgccactcaat |  | R for complementation of Δ*CsuAB* (HindIII) |
| *pilA*-up-F | cggtgattcagtcctact | 194 | F for upstream of Δ*PilA* |
| *pilA*-up-R | gccttttccccaaagattg |  | R for upstream of Δ *PilA* |
| *pilA*-down-F | gagagaggtttcgacctc | 171 | F for downstream of Δ*PilA* |
| *pilA*-down-R | agtgctaaaggaaactgc |  | R for downstream of Δ*PilA* |
| *pilA*-kan-F | caatctttggggaaaaggcgtgtaggctggagctgcttc | 1509 | F for Δ*PilA* kan cassette |
| *pilA*-kan-R | gaggtcgaaacctctctcccatatgaatatcctccttag |  | R for Δ*PilA* kan cassette |
| *pilA*-com-F | ccctttcgtcttcaagaattcgaattaggtaacgcacct | 847 | F for complementation of Δ*PilA* (EcoRI) |
| *pilA*-com-R | taaactaccgcattaaagcttgaggtcgaaacctctctc |  | R for complementation of Δ*PilA* (HindIII) |
| CR-sp20 seed F | AATTCgttacgtctacggtacgtggttagcatgatttccA | 32 | F for the protospacer of CR-sp20 (EcoRI) |
| CR-sp20 seed R | AGCTTaaatcatgctaaccacgtaccgtagacgtaacggG |  | R for the protospacer of CR-sp20 (HindIII) |
| CR-sp20 M F | AATTCgttacgtctacggtacgtggttagcatCatttccA | 32 | F for the protospacer of CR-sp20 with a one-base seed mutation (EcoRI) |
| CR-sp20 M R | AGCTTaaatGatgctaaccacgtaccgtagacgtaacggG |  | R for the protospacer of CR-sp20 with a one-base seed mutation (HindIII) |
| CR-sp20 check F | cagaccgaacacttctaaa | 143 |  |
| CR-sp20 check R | acccaagcggtgaattaac |  |  |
| CR-sp50 seed F | AATTCggtttaagtccccgaacattacataagtaaatccA | 32 | F for the protospacer of CR-sp50 (EcoRI) |
| CR-sp50 seed R | AGCTTatttacttatgtaatgttcggggacttaaaccggG |  | R for the protospacer of CR-sp50 (HindIII) |
| CR-sp50 M F | AATTCggtttaagtccccgaacattacataaCtaaatccA | 32 | F for the protospacer of CR-sp50 with a one-base seed mutation (EcoRI) |
| CR-sp50 M R | AGCTTatttaGttatgtaatgttcggggacttaaaccggG |  | R for the protospacer of CR-sp50 with a one-base seed mutation (HindIII) |
| CR-sp50 check F | ctgtaaaacagaagacttgcc | 142 |  |
| CR-sp50 check R | gaaatccccgtcagcagcaaat |  |  |
| CR-sp75 seed F | AATTCtatttgctacgccaccagccatacctagaagcccA | 32 | F for the protospacer of CR-sp75 (EcoRI) |
| CR-sp75 seed R | AGCTTgcttctaggtatggctggtggcgtagcaaataggG |  | R for the protospacer of CR-sp75 (HindIII) |
| CR-sp75 M F | AATTCtatttgctacgccaccagccatacctaCaagcccA | 32 | F for the protospacer of CR-sp75 with a one-base seed mutation (EcoRI) |
| CR-sp75 M R | AGCTTgcttGtaggtatggctggtggcgtagcaaataggG |  | R for the protospacer of CR-sp75 with a one-base seed mutation (HindIII) |
| CR-sp75 check F | atagcttcttccaactac | 141 |  |
| CR-sp75 check R | attcggctgtgccttttatg |  |  |
| 16sRNA-F | gttgtggctttaggtttattatacg |  |  |
| 16sRNA-R | aagttactcgacgcaattcg |  |  |
| q-*cas3*-F | gccaagacttgattgcgattgcc |  |  |
| q-*cas3*-R | taacgaccaaccgtgctgataagc |  |  |
| q-*h-ns*-F | cagcaactaaccaacgtgg |  |  |
| q-*h-ns*-R | aacttctagaatttggcgctc |  |  |
| q-*baeR*-F | gtcaggatgcttacacgagc |  |  |
| q-*baeR*-R | ctgttcacgtaccttacggc |  |  |
| q-*csuAB*-F | tacggcttctgctgatgtag |  |  |
| q-*csuAB*-R | accgaaaattggtacggcag |  |  |
| q-*fimD*-F | accttgggctaccattcc |  |  |
| q-*fimD*-R | caacaaaaacgggatatgcag |  |  |
| q-*pilA*-F | gcccgatctcaaatgtcag |  |  |
| q-*pilA*-R | ctggaataccatttgctccag |  |  |
