# Supplementary material for: BaeR and H-NS control CRISPR-Cas-mediated immunity and virulence in Acinetobacter baumannii
Source: mSystems. 2025 Oct 31;10(11):e01067-25. doi: 10.1128/msystems.01067-25 (PMC12625773; doi:10.1128/msystems.01067-25)
Supplement: Table S2 — Identification of proteins to the cas3 promoter region by MS. [file msystems.01067-25-s0007.docx]

Table S2: Identification of proteins to the *cas3* promotor region by MS.

| Reference | Protein | Locus tag |
| --- | --- | --- |
| tr\|D0C9P8\|D0C9P8_ACIB2 Elongation factor G | FusA | K9O37_14240 |
| tr\|D0C5Z7\|D0C5Z7_ACIB2 Phosphoenolpyruvate synthase | ppsA | K9O37_11640 |
| tr\|D0CBU4\|D0CBU4_ACIB2 Isocitrate dehydrogenase |  | K9O37_04910 |
| sp\|A3M1F6\|EFTU_ACIBT Elongation factor Tu | tuf | K9O37_14235 |
| tr\|D0CEK6\|D0CEK6_ACIB2 ATP synthase subunit alpha | atpA | K9O37_17840 |
| tr\|D0CDF8\|D0CDF8_ACIB2 50S ribosomal subunit assembly factor BipA | typA | K9O37_03205 |
| tr\|D0CAU8\|D0CAU8_ACIB2 Polyribonucleotide nucleotidyltransferase | pnp | K9O37_16850 |
| tr\|D0CEK4\|D0CEK4_ACIB2 ATP synthase subunit beta | atpD | K9O37_17830 |
| tr\|A0A7U3Y891\|A0A7U3Y891_ACIB5 NADP-dependent malic enzyme |  | K9O37_05540 |
| tr\|A0A7U4DHH7\|A0A7U4DHH7_ACIB5 Isocitrate dehydrogenase | icd | K9O37_04920 |
| tr\|A0A7U4DH05\|A0A7U4DH05_ACIB5 Dihydrolipoyllysine-residue succinyltransferase component of 2-oxoglutarate dehydrogenase complex | odhB | K9O37_03890 |
| sp\|B7IA36\|RL2_ACIB5 Large ribosomal subunit protein uL2 | rplB | K9O37_02225 |
| tr\|A0A7U4DFP2\|A0A7U4DFP2_ACIB5 Phenylalanine--tRNA ligase beta subunit | pheT | K9O37_15310 |
| tr\|A0A7U4DFI5\|A0A7U4DFI5_ACIB5 DUF3298 domain-containing protein |  | K9O37_15710 |
| sp\|B7IBK5\|DNAK_ACIB5 Chaperone protein DnaK | dnaK | K9O37_18540 |
| tr\|A0A8I1ZHC8\|A0A8I1ZHC8_ACIBA Serine hydroxymethyltransferase |  | K9O37_06090 |
| tr\|A0A7U3Y8I2\|A0A7U3Y8I2_ACIB5 Electron transfer flavoprotein subunit beta |  | K9O37_04360 |
| tr\|D0CFC1\|D0CFC1_ACIB2 Phosphoribosylaminoimidazole-succinocarboxamide synthase |  | K9O37_00295 |
| tr\|A0A7U3Y9R3\|A0A7U3Y9R3_ACIB5 Porin |  | K9O37_00890 |
| tr\|D0CD03\|D0CD03_ACIB2 30S ribosomal protein S3 | rpsC | K9O37_02240 |
| tr\|A0A7U4DH43\|A0A7U4DH43_ACIB5 succinate dehydrogenase | sdhB | K9O37_03870 |
| tr\|Q4A209\|Q4A209_ACIBA Putative outer membrane protein | caro | K9O37_04635 |
| tr\|D0C5K7\|D0C5K7_ACIB2 ferredoxin--NADP(+) reductase |  | K9O37_12480 |
| tr\|D0CA65\|D0CA65_ACIB2 Acetyl-coenzyme A carboxylase carboxyl transferase subunit alpha | accA | K9O37_15275 |
| tr\|D0CD21\|D0CD21_ACIB2 30S ribosomal protein S4 | rpsD | K9O37_02330 |
| tr\|A0A7U3Y580\|A0A7U3Y580_ACIB5 Protein tolA |  | K9O37_06395 |
| tr\|A0A6H3EAZ4\|A0A6H3EAZ4_ACIBA Histone H2A |  | K9O37_17320 |
| tr\|A0A654L0V5\|A0A654L0V5_ACIBM Peptidyl-prolyl cis-trans isomerase |  | K9O37_18315 |
| tr\|A0A654KZE1\|A0A654KZE1_ACIBM Universal stress protein |  | K9O37_06735 |
| tr\|A0A654KXZ7\|A0A654KXZ7_ACIBM cAMP-activated global transcriptional regulator CRP | CRP | K9O37_06305 |
